# Lake productivity and waterbird functional diversity across geographic and environmental gradients in temperate China

**DOI:** 10.1002/ece3.6763

**Published:** 2020-09-23

**Authors:** Yamian Zhang, Wenzhuo Tan, Qing Zeng, Haitao Tian, Yifei Jia, Guangchun Lei, Li Wen

**Affiliations:** ^1^ School of Ecology and Nature Conservation Beijing Forestry University Beijing China; ^2^ College of the Environment & Ecology Xiamen University Xiamen China; ^3^ Science, Economics and Insights Division Department of Planning, Industry and Environment Lidcombe NSW Australia

**Keywords:** climate, geographical gradients, inland wetlands, lake size, structural equation model

## Abstract

Geographical gradients in species diversity have long fascinated biogeographers and ecologists. However, the extent and generality of the effects of the important factors governing functional diversity (FD) patterns are still debated, especially for the freshwater domain. We examined the relationship between lake productivity and functional diversity of waterbirds sampled from 35 lakes and reservoirs in northern China with a geographic coverage of over 5 million km^2^. We used structural equation modeling (SEM) to explore the causal relationships between geographic position, climate, lake productivity, and waterbird FD. We found unambiguous altitudinal and longitudinal gradients in lake productivity and waterbird FD, which were strongly mediated by local environmental factors. Specifically, we found (a) lake productivity increased northeast and decreased with altitude. The observed geographic and altitudinal gradients were driven by climatic conditions and nutrient availability, which collectively explained 93% of the variations in lake productivity; (b) waterbird FD showed similar geographic and altitudinal gradients; the environmental factors which had direct and/or indirect effects on these gradients included climate and lake area, which collectively explained more than 39% of the variation in waterbird FD; and 3) a significant (*p* = .029) causality between lake productivity and waterbird FD was confirmed. Nevertheless, the causality link was relatively weak in comparison with climate and lake area (the standardized path coefficient was 0.55, 0.23, and 0.03 for climate, lake area, and productivity, respectively). Our study demonstrates how the application of multivariate technique (e.g., SEM) enables the illustration of complex causal paths in ecosystems, enhancing mechanistic explanations that underlie the observed broadscale biodiversity gradients.

## Graphical abstract

The article reveals the key environmental drivers of large‐scale patterns in freshwater lake productivity and waterbird functional diversity in the arid and semi‐arid northern China.

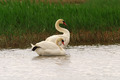

## 1 INTRODUCTION

The planet Earth shows striking gradients in the diversity of plants and animals, from high biodiversity in the tropics to low biodiversity in polar and high‐mountain regions (Gaston, 2000; Rosenzweig, 1995; Whittaker, Nogues‐Bravo, & Araujo, 2007; Willig, Kaufman, & Stevens, 2003). Due to the alarming rate of biodiversity loss in the last decades caused by anthropogenic interruption (Prescott et al., 2016; Waide et al., 1999), renewed interests in taxonomic diversity patterns are likely to contribute to important insights for developing a more general theory of species diversity (Castro‐Insua, Gómez‐Rodríguez, & Baselga, 2016). Studies in the past decades have explored the mechanisms for such patterns, leading to conceptual insights on the biogeographical variation of species diversity (Devictor et al., 2010; Field et al., 2009; Gaston, 2000). For example, diversity is often highest at intermediate levels of ecosystem productivity (Grime, 1973; Mittelbach et al., 2001; Waide et al., 1999), and species diversity increases with habitat area (MacArthur & Wilson, 1967; Rosenzweig, 1995) and/or habitat patches, for environmental heterogeneity is considered as a key driver of species diversity across taxa, biomes, and spatial scales (Stein, Gerstner, & Kreft, 2014). In addition, both theoretical considerations and empirical analyses suggest that the spatial patterns of species diversity are likely scale dependent (Field et al., 2009; Mittelbach et al., 2001; Mouchet et al., 2015).

Most macroecology research has been focused on terrestrial ecosystems (Currie & Paquin, 1987; Qian, Ricklefs, & White, 2005), and relative fewer studies have explored geographic biodiversity gradients and the underling mechanisms for aquatic ecosystems (Astorga, Heino, Luoto, & Muotka, 2011; Barbour & Brown, 1974; Heino, 2002, 2011; Irz, Argillier, & Thierry, 2004; Jacobsen, 2004), especially the aquatic ecosystems in the arid and semi‐arid region, which provide important habitats for diverse species and water resources for human living (Williams, 1999). Moreover, the geographical distribution of study sites is strongly biased toward Europe and North America, with particularly poor coverage in Asia (Field et al., 2009; Fu, Wu, Wang, Lei, & Chen, 2004). Study of species diversity in aquatic ecosystems is as essential as in their terrestrial counterparts (Stendera et al., 2012). Declines in biodiversity are far greater in freshwaters than in most terrestrial ecosystems (Sala et al., 2000). Freshwater ecosystems are one of the most endangered ecosystems in the world (Dudgeon et al., 2006; Millennium Ecosystem Assessment, 2005), and the actual rates of freshwater species extinction due to human interruptions are much higher than natural extinction rates (Naiman & Dudgeon, 2011). Therefore, a better understanding of the global freshwater diversity gradients and the major environmental drivers remains a major topic (Heino, 2011); and such studies serve to address some fundamental questions for the conservation of freshwater taxa (Tisseuil et al., 2013).

**FIGURE 1:**
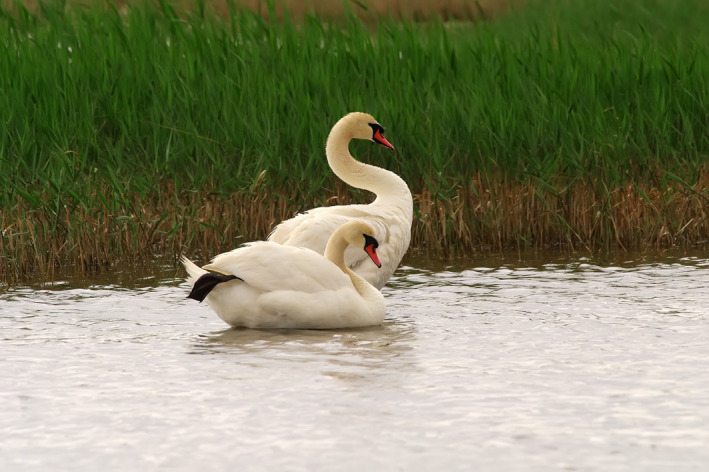
A pair of Mute Swans (*Cygnus olor*) inhabits in Wuliangsuhai Lake, Inner Mongolia

Waterbirds are ubiquitous components of freshwater systems, and their diversity and abundance have long been recognized as suitable bioindicators of environmental change in aquatic systems (Caro & O'Doherty, 1999; Wen, Saintilan, Reid, & Colloff, 2016) and serve multiple significant functional roles in ecosystems (Barbet‐Massin & Jetz, 2015) (Figure 1). Worldwide, strong geographic differences exist in the ecological attributes of birds (Kissling, Sekercioglu, & Jetz, 2012). However, as with other freshwater biota, macroecological studies of environmental drivers of waterbird diversity are rare (Shah, Domisch, Pauls, Haase, & Jähnig, 2014; Stendera et al., 2012; Zeng et al., 2019). It is unclear whether similar latitudinal and other broad geographical gradients (e.g., altitudinal) apply to waterbirds as well. In a recent review, Heino (2011) found no clear latitudinal gradients at regional scale while species richness typically attains highest levels in mountainous regions. Using river basins as the spatial unit, however, Tisseuil et al. (2013) found that the ‘climate/productivity’ hypothesis (Field et al., 2009) explained large portion of geographic variance in waterbird richness, which is consistent to land avian species (Storch et al., 2006). Several factors are known to affect waterbird diversity at a local scale, such as lake productivity, lake size, and habitat heterogeneity (Barbour & Brown, 1974; Cintra, 2015; Xia et al., 2016). For example, lake productivity is often a strong predictor of freshwater biodiversity (Dodson, Arnott, & Cottingham, 2000), including aquatic animals (and zooplankton as well) (Chase & Leibold, 2002) and phytoplankton (Stomp, Huisman, Mittelbach, Litchman, & Klausmeier, 2011). But its effect on waterbirds has rarely been tested. The productivity–diversity hypothesis suggests a positive effect of primary productivity on species diversity by allowing larger populations to persist, thereby reducing extinction risk and supporting a higher diversity of niche specialists (Tittensor et al., 2010; Willig et al., 2003). Linking these local scale variables with broadscale geographical variations in an integrative analysis framework could potentially articulate the leading processes underlying the regional and global waterbird diversity patterns.

Biodiversity assessment is an important component of conservation planning and increasingly used to identify land‐use management practices that maximize both evolutionary value and ecosystem function (Chapman, Tobias, Edwards, Davies, & Vamosi, 2018). Key requirements are to maintain community resilience to environmental disturbance and to preserve ecosystem functions and services across time and space (Socolar, Gilroy, Kunin, & Edwards, 2016). Consequently, it is often proposed that we need to look beyond merely conserving species richness toward maintaining the maximum diversity of evolutionary lineages and associated ecological functions (Bregman et al., 2016). The idea that functional diversity (FD) or functional complementarity performs better than species richness as predictors of ecosystem functions are supported by a range of empirical studies (Flynn, Mirotchnick, Jain, Palmer, & Naeem, 2011; Fründ, Dormann, Holzschuh, & Tscharntke, 2013; Petchey & Gaston, 2007). FD is a biodiversity component that represents the extent of the functional differences among species based on the distinction of their morphological, physiological, and ecological traits (Petchey & Gaston, 2006). Species loss may lead to a reduction in FD depending on the intrinsic redundancy of assemblages (Flynn et al., 2009; Petchey, Evans, Fishburn, & Gaston, 2007). A decrease on the FD of local and regional assemblages could have dramatic consequences for ecosystem functioning because the traits of species, not just the number of taxonomic units, ultimately drive biodiversity–ecosystem functioning relationships (Díaz & Cabido, 2001; Hooper et al., 2005).

In this case, we applied FD metrics to waterbird communities sampled in 35 lakes across the entire temperate arid and semi‐arid northern China to (a) explore the geographical patterns of waterbird diversity and lake productivity, and their influencing factors; (b) test the productivity–diversity hypothesis; and more importantly, (c) fill in the gaps in our understanding of ecological patterns in aquatic ecosystems across a large geographical scale.

## 2 MATERIALS AND METHODS

### 2.1 Study sites

**FIGURE 2:**
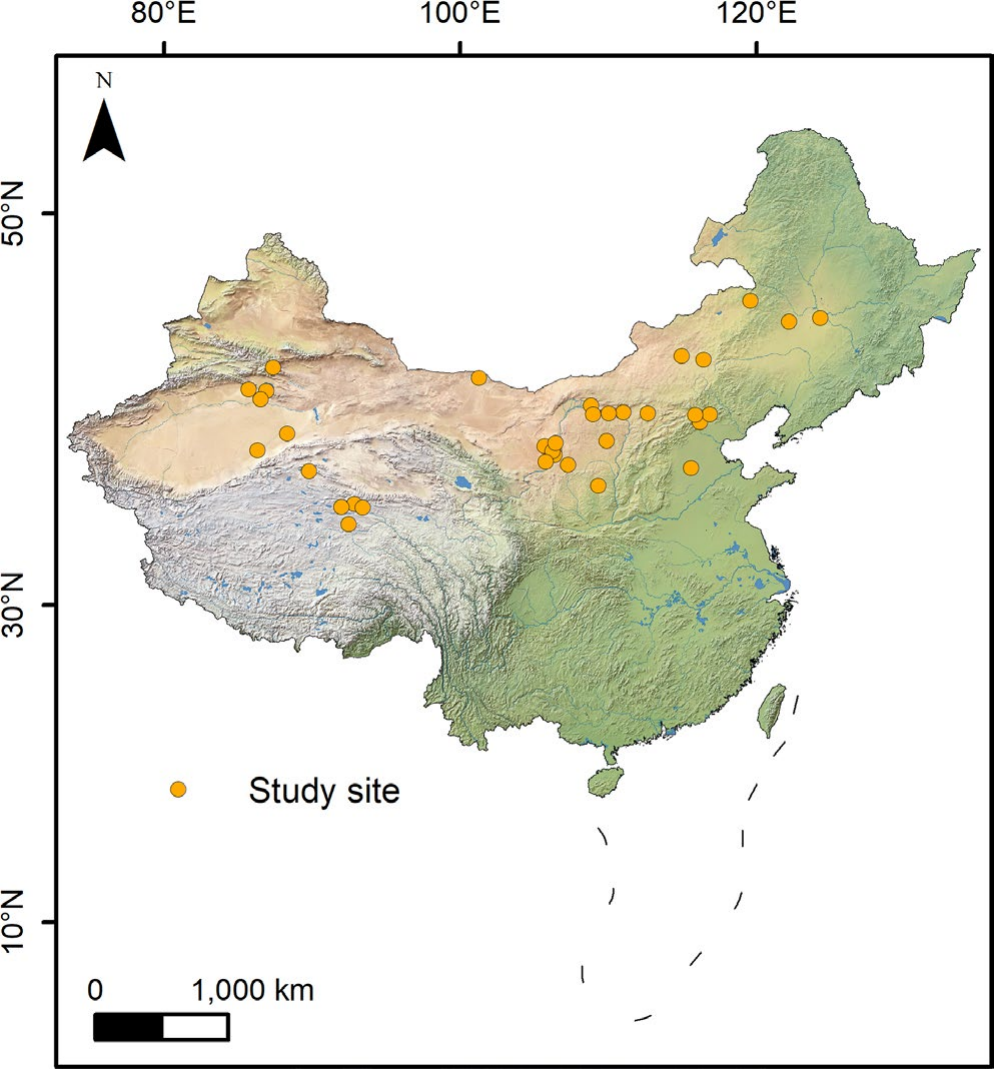
Locations of the 35 surveyed lakes and reservoirs in North China. Elevation gradient is used as background

We sampled a total of 35 lakes and reservoirs across the temperate zone of China (latitude 34.60° to 46.06°, longitude 85.69° to 124.29°, Figure 2). The study area covers more than 5 million km^2^, including a range of landforms such as mountains, hills, plateaus, and plains, with altitude ranging from 22.61 m to 4,818.10 m. This area has large precipitation and temperature gradients. The surveyed waterbodies show a range of physical, chemical, and topographic characteristics (Table S1). Most of the lakes are located in the arid and semi‐arid region of China, providing critical habitats for diverse species including waterbirds. Human uses of the lakes mainly include fishery, tourism, water source for irrigation, and reed harvest.

### 2.2 Data collection

Data were collected from 35 lakes and reservoirs during the summers of 2011–2016. The lakes were sampled one to three times, with most of them sampled once in summer. In each lake, water samples were taken on the same day as waterbird survey during daylight hours.

#### 2.2.1 Waterbird surveys

Waterbirds were surveyed using transect line method to maximize the spatial coverage of the lakes. Most of the transects were set in the lake shore, while in some lakes with larger area (e.g., Boston Lake in Xinjiang Province and Wuliangsuhai Lake in Inner Mongolia), transects were also set in lakes (open water area). Fixed transects with variable lengths were established in each lake and reservoir. Based on the size of each study site, the transect length varied from 1 to 5 km for the lake shore transects and 8 to 13 km for transects in lakes. The perpendicular searching distance varied from 0.1 to 0.6 km. We surveyed the lake shore transects on foot at a constant speed, whereas boats with low and constant velocity were used for the transects in lakes. All the transect lines were surveyed by using binoculars (8 × 42) and telescopes (Swarovski ATS 80 HD 20–60 × 80). To increase detectability, the surveys were carried out on clear days during daytime and there were at least two fully trained observers for each transect. Visual and/or verbal communication enabled us to avoid duplicate recordings of the same flock of waterbirds by the observers. Waterbirds were all identified to species level.

We acknowledge that the above surveys may be insufficient to detect all species in a site because of the high mobility of waterbirds. Thus, historical waterbird surveys, either by the nature reserves or published, studies were also collected and incorporated. On 31 December 2017, we conducted a search of all peer‐reviewed indexed in the scientific database ISI Web of Science‐SCI‐Expanded and CNKI (for Chinese publications). The keywords used include the names of each lake and reservoir (e.g., Wuliangsuhai) and the names of the study regions (e.g., Beijing, Inner Mongolia, respectively) plus “bird*” or “waterbird*” or “waterfowl*” or “avian*”. In addition, other gray literatures like books and reports were collected. Information of waterbird species at each study site was extracted from the publications. Waterbird lists from each nature reserve were carefully checked and consulted with ornithologists, and nature reserve staffs when necessary to make sure the records are correct. To incorporate those data with our survey results, only waterbird species that recorded during 2011 to 2016 (our sampling period) were used.

#### 2.2.2 Functional trait data

To estimate functional diversity, we collected biometric trait data that describe key ecological attributes of species from published literature for all 148 species. We collected 16 traits for each bird species, including generation length (the average age of breeding individuals), clutch size (number of eggs), incubation times, body size, body mass, wingspan, migratory types (full migration, altitudinal migration, and not migration), breeding habitat range, diet (percentage of scavenger, and invertebrate, fish, seed, fruit, and other plant materials), habitat mode (percentage usage of water, riparian, and ground), and pelagic species (0/1) (Table 1). These selected traits measure many aspects of resource used by birds, such as the quantity and the quality of resource consumed (Petchey & Gaston, 2007), as well as the fitness of the species such as reproduction strategies and generation length (Luck, Andrew, Lisa, & Davies, 2013). For example, body mass is highly related to birds’ energy requirements (Blendinger & Villegas, 2011). Diet is related to ecosystem functions such as seed dispersal and food‐web structure (Sekercioglu, 2006). The main sources of the trait measurements are Planet of Birds, BirdLife International, and a database compiled by Wilman et al. (2014) (Table 1). Any missing data were filled based on information in the ornithological literature, such as the Handbook of the Birds of the World (http://www.hbw.com/). A brief summary is available in Table S2.

**TABLE 1:** The five trait types and 16 specific traits used to characterize waterbird functional diversity in lakes and reservoirs located in North China

Type	Trait	Mean	*SD*	Sources
Resource quantity	Size (cm)	48.48	31.65	a and b
	Mass (g)	1,078.07	1912.80	a and b
	Wingspan (cm)	89.86	52.22	a
	Migration	–	–	b
	Pelagic	–	–	b
	Breeding range (km^2^, log‐transformed)	7.24	0.71	b
Life history	Generation (year)	8.07	2.85	a
Reproduction	Clutch size (no. of eggs)	4.55	2.17	a and b
	Incubation (day)	24.89	4.63	c
Diet	Diet‐Inv (% of invertebrate)	49.53	31.84	c
	Diet‐V (% of vertebrate)	22.84	28.17	c
	Diet‐Scav (% of scavenger)	0.88	3.84	c
	Diet‐Plant (% of plant materials)	26.76	31.97	c
Habitat	Hab‐W (% of time on water surface)	15.20	30.87	c
	Hab‐Rip (% of time on riparian area)	40.34	28.88	c
	Hab‐G (% of time on ground)	41.91	32.51	c

Note

(a) Planet of Birds, website visited 23 June 2016: http://www.planetofbirds.com/. (b) BirdLife International, website visited 28 June 2016: http://datazone.birdlife.org/. (c) Wilman et al., 2014.

We used functional richness (FRic) and functional dispersion (FDis) as the measure of functional diversity. FRic represents the amount of functional space filled by the community (Villéger, Mason, & Mouillot, 2008). A community with high FRic would be one with many traits (and potentially high utilization of resources), whereas one with lower FRic might indicate that some niches are not available in the ecosystem (Prescott et al., 2016). FDis is the mean distance in multidimensional trait space of individual species to the centroid of all species (Laliberté & Legendre, 2010), which is influenced both by the range of trait values and the distribution of individuals within trait space (Prescott et al., 2016) but not related mathematically with species richness (Laliberté & Legendre, 2010). Large values of FDis imply that many species have long distances to the community centroid, indicating communities with many specialist species and high potential for species’ complementarity (Laliberté & Legendre, 2010).

#### 2.2.3 Lake chemical characteristics and size

Indicators for lake chemical characteristics include total nitrogen (TN), total phosphorus (TP), and chlorophyll *a* (Chl‐*a*). Water samples for TN and TP in each lake were collected and preserved using 100‐ml jars on the same day with waterbirds surveys. All the water samples were sent to and processed in laboratory by using Ultrospec 6300 pro spectrophotometer (GE Healthcare, America). Water samples for chlorophyll *a* (Chl‐a) concentration were collected synchronously on site. We used 2‐liter bottles to collect water samples in each lake and reservoir and then filtered the water by using GF/C filter membrane, each filter membrane was filtered with 500 ml water, and three filter membranes (3 × 500 ml) were requested for every sampling point. All water samples and filter membranes were preserved in <5℃ refrigerator and sent to laboratory for further test by using Ultrospec 6300 pro spectrophotometer (GE Healthcare, America). Sample size ranges from 3 to 18 based on the area of lake. We set three, six, nine, 12, and 18 sampling points for lakes with area <10,000 ha, 10,000–20,000 ha, 20,001–30,000 ha, 30,001–50,000 ha, and >50,000 ha, respectively. Surface area of each lake and reservoir was calculated from remote sensing interpretation by using Google Earth images from year 2013 to 2016.

#### 2.2.4 Climatic and geographical variables

To define the climate of the lakes, we used the 30 s WorldClim bioclimatic variables, which were downloaded from WorldClim website, publicly available at https://www.worldclim.org/data/worldclim21.html. The following bioclimatic variables were included in the study based on collinearity test (see below): mean diurnal range (T1, mean of monthly (max temp‐min temp)), mean temperature of the wettest quarter (T2), mean temperature of the warmest quarter (T3), annual total precipitation (P1), precipitation of the driest month (P2), precipitation of the warmest quarter (P3), and precipitation of the coldest quarter (P4). The variables of climate are spatial means of each lake. Altitudes of the sampled lakes were retrieved from GPS Visualizer on site. Mean geographical coordinates of each site were calculated from remote sensing interpretation by using Google Earth images of each lake and reservoir between years 2013 to 2016.

### 2.3 Data analyses

#### 2.3.1 Data preparation

We combined our own survey data and historical records to compile a waterbird community dataset for each lake. FD was then calculated based on the compiled community dataset. It is necessary to have several years of observation because the species observed in a lake could vary from year to year. In addition, we used the total chlorophyll *a* proxy of primary productivity (Eppley, Stewart, Abbott, & Heyman, 1985; Falkowski & Raven, 2013).

Environmental variables were log_10_‐transformed if this resulted in a more uniform spread of data points. We checked the collinearity of all environmental variables based on variance inflation factor by using the VIF function in R package “*car*” (Fox et al., 2012). All variables with VIF greater than 10 were excluded.

#### 2.3.2 Structural equation modeling—exploring the drivers of lake productivity and waterbird functional diversity

We used the structural equation modeling (SEM) framework to investigate the causal links between environmental gradients, lake productivity, and waterbird FD. SEM is a collection of procedures whereby complex hypotheses, particularly those involving networks of path relations, are evaluated against multivariate data (Grace, 2006). The resulting estimates for path coefficients in SEM represent the implied sensitivities of response variables to variations in individual predictors (Grace et al., 2012). It is a system of linear equations among several unobservable variables (latent factors) and observed variables (indicator variables). The latent factors are variables that are unobserved, but whose influence can be summarized through one or more indicator variables. They are useful for capturing complex or conceptual properties of a system that difficult to quantify or measure directly. We started with an SEM that included all plausible pathways between waterbird FD, the selected set of local environmental variables, and the geographical coordinates of the lakes (Figure S1). Our initial attempt revealed that the model was under‐identified, meaning that there was some redundancy such that it was not possible to estimate all the model's parameters. We therefore investigated the statistical relationships among the variables included in the model to identify possible redundancies. Subsequently, we fitted two individual SEMs separately for lake productivity and waterbird FD, and an integrated SEM to explore the causal link between lake productivity and waterbird FD.

These SEMs had similar hierarchical structure, in which the responsible variable (i.e., lake productivity or waterbird FD) was modeled by a few latent factors. These latent factors were in term defined by the observed variables. In all models, two latent factors were common: geographic position and climate. The geographic position was measured by the latitude and longitude of the central point of the lake and its average elevation. The climate was defined by using spatial mean of the selected bioclimatic variables. A causality from geographic position to climate condition was specified in all SEMs. In addition, in the productivity SEM, nutrient level (defined by TN and TP) was also included in the model as latent variable; while in the waterbird FD SEM, lake size (defined solely by lake area) was analyzed as lake surface area plays an important role in driving waterbird diversity (Murphy & Dinsmore, 2018; Pescador, Díaz, & Peris, 2012; Zhao & Zhou, 2018).

We tested the significance of each path coefficient using 1,000 bootstrapped resamples and reported the standardized path coefficients that can be directly compared to make inferences about the relative strength of relationships (Grace & Bollen, 2005). The structural equation analyses were performed using R version 3.6.1 (R Development Core Team, 2019) with package “Lavaan” version 3.1‐3 (Rosseel, 2012).

## 3 RESULTS

### 3.1 Spatial patterns of waterbird communities and lake productivity

**FIGURE 3:**
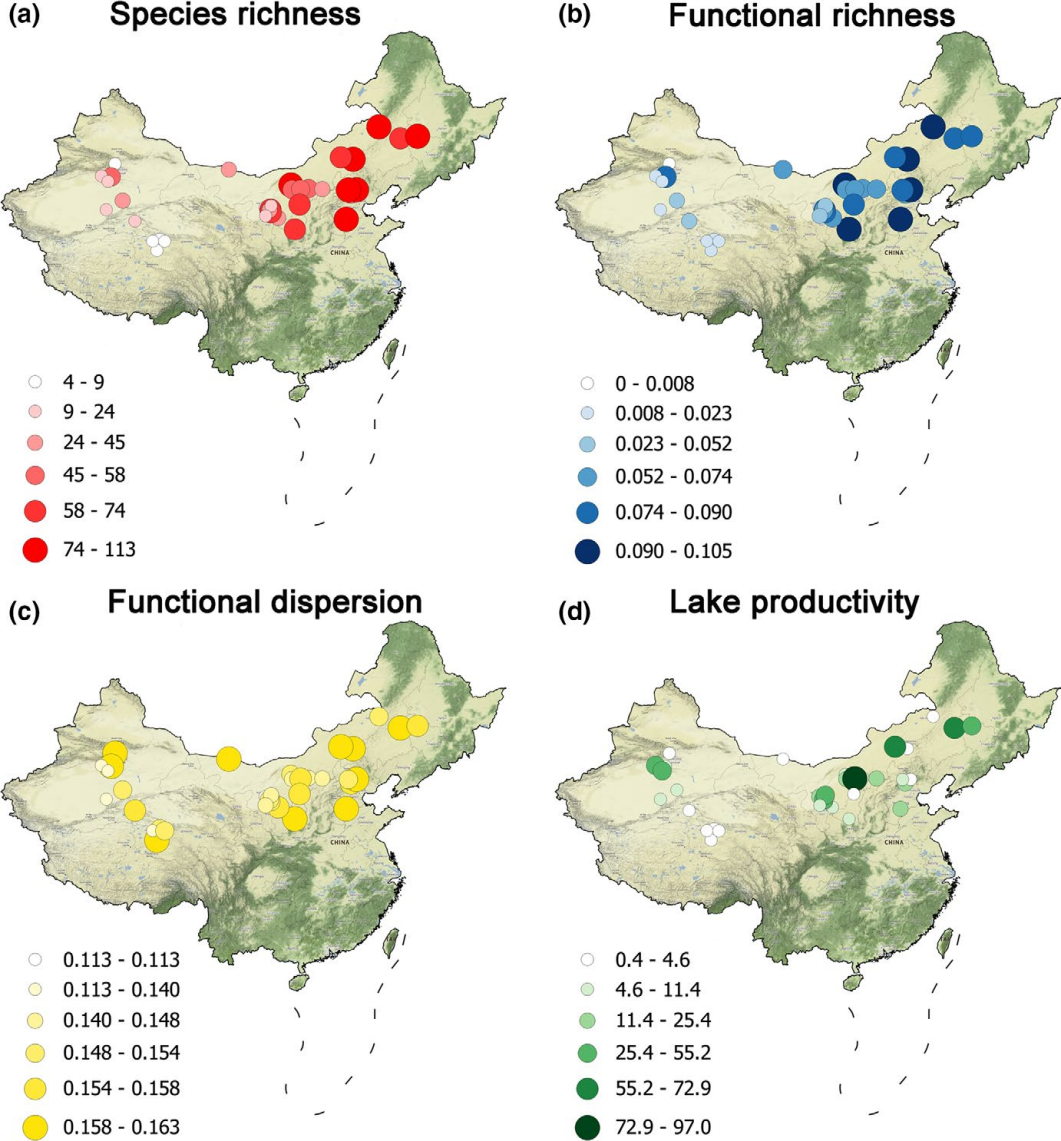
Map of the 35 surveyed lakes and reservoirs in North China showing (a) waterbird species richness, (b) functional richness, (c) functional dispersion, and (d) lake productivity (mg/m^3^)

A total of 148 species, belonging to six orders and 19 families, were recorded in this study (Figure S2). The highest number of species was from the family Scolopacidae (37 species), followed by Anatidae (34 species). Families of Phalacrocoracidae, Threskiornithidae, and Rostratulidae only had one species each. Waterbird richness varied greatly among the 35 lakes and reservoirs surveyed, ranging from 4 to 113 species (Figure 3a).

Generally, the higher species richness was recorded in lakes and reservoirs located in the more humid northern China Plain (e.g., Hubei Province and Beijing) than in the drier western China such as Inner Mongolia and Qinghai Province. Also, an overall decreasing altitudinal gradient in species richness was observed (Figure 3a). While waterbird FRic showed similar pattern as species richness (Figure 3b), FDis had no clear spatial pattern and was not correlated with species richness (Figure 3c). Like waterbird richness and FRic, lake productivity showed a clear altitudinal gradient. However, the latitudinal and longitudinal gradients were more obscure (Figure 3d).

### 3.2 Determinates of lake productivity

**FIGURE 4:**
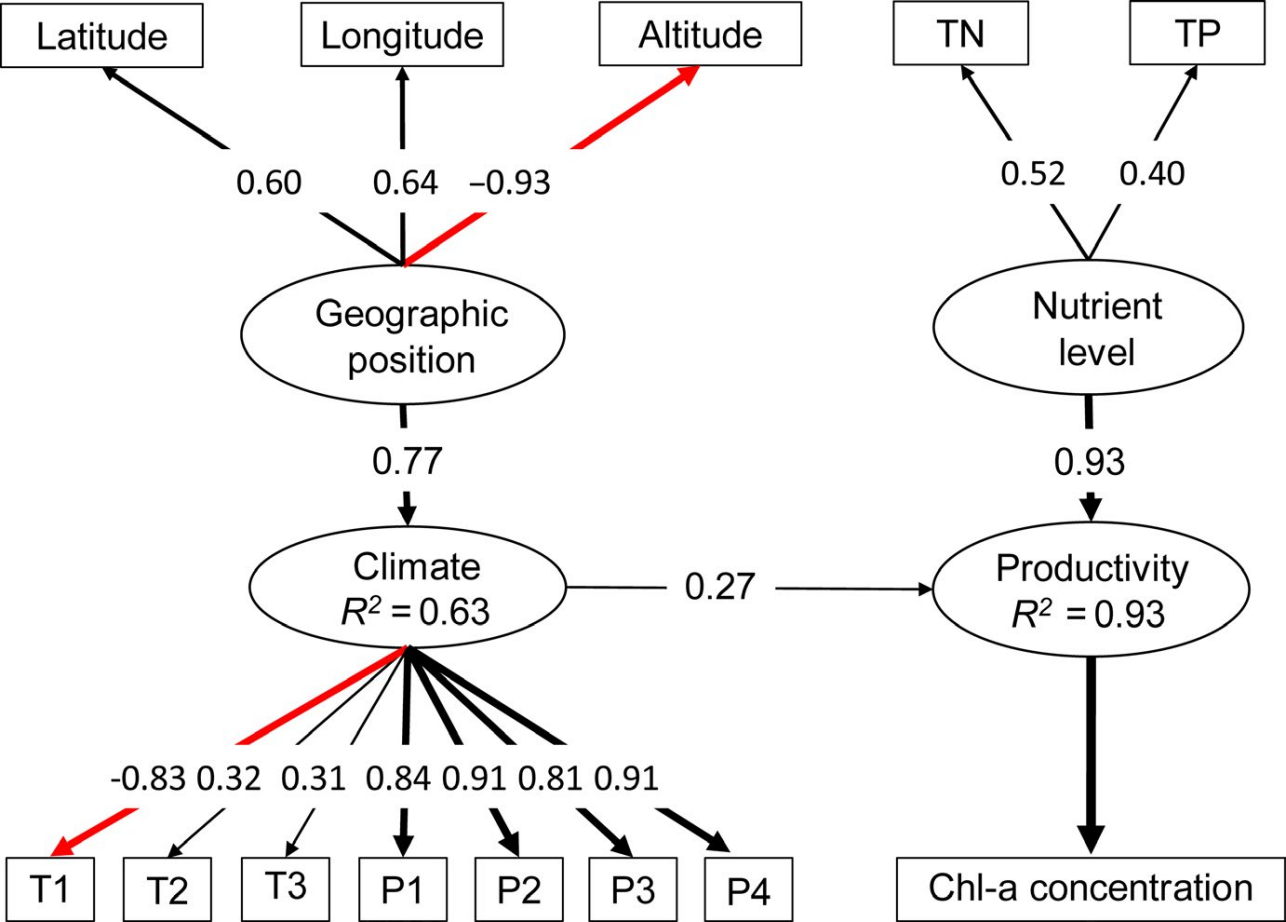
SEM for lake productivity. Latent variables are in ovals and measured variables in rectangles. The strength of the causality (standardized path coefficient) is indicated by the width of the line. Black lines indicate positive effects, while red lines mean negative impacts. T1: mean diurnal range, T2: mean temperature of the wettest quarter, T3: mean temperature of the warmest quarter, P1: annual total precipitation, P2: precipitation of the driest month, P3: precipitation of the warmest quarter, and P4: precipitation of the coldest quarter

Three latent factors (i.e., geographic position, climate, and nutrient level) were all included in the SEMs. The three factors collectively explained 93% of the variation in lake productivity (Figure 4). Lake nutrient level, which was defined by water TP and TN concentration, had the largest effects on productivity, followed by climate (standardized path coefficient = 0.93 and 0.77 for nutrient and climate, respectively, Figure 4). The effects of geographic position on lake productivity were indirect and realized through its influence on climatic condition, which had a standardized path coefficient of 0.16 (0.77 × 0.21).

Lake productivity decreased with elevation (path coefficient = −0.93 × 0.77 × 0.07 = −019) and increased with both latitude and longitude with comparable path coefficient (0.12 and 0.13 for latitude and longitude, respectively, Figure 4). For the measured bioclimatic variables, while all variables related to precipitation as well as T2 and T3 had positive effect on lake productivity, the impact of temperature diurnal range (T1) was negative with a path coefficient of −0.22 (−0.83 × 0.27). Lake productivity was positively related with nutrient; and the effect of TP level was slightly larger than that of TN (standardized path coefficient = 0.52 and 0.40 for TP and TN, respectively, Figure 4).

### 3.3 Determinates of waterbird functional diversity

**FIGURE 5:**
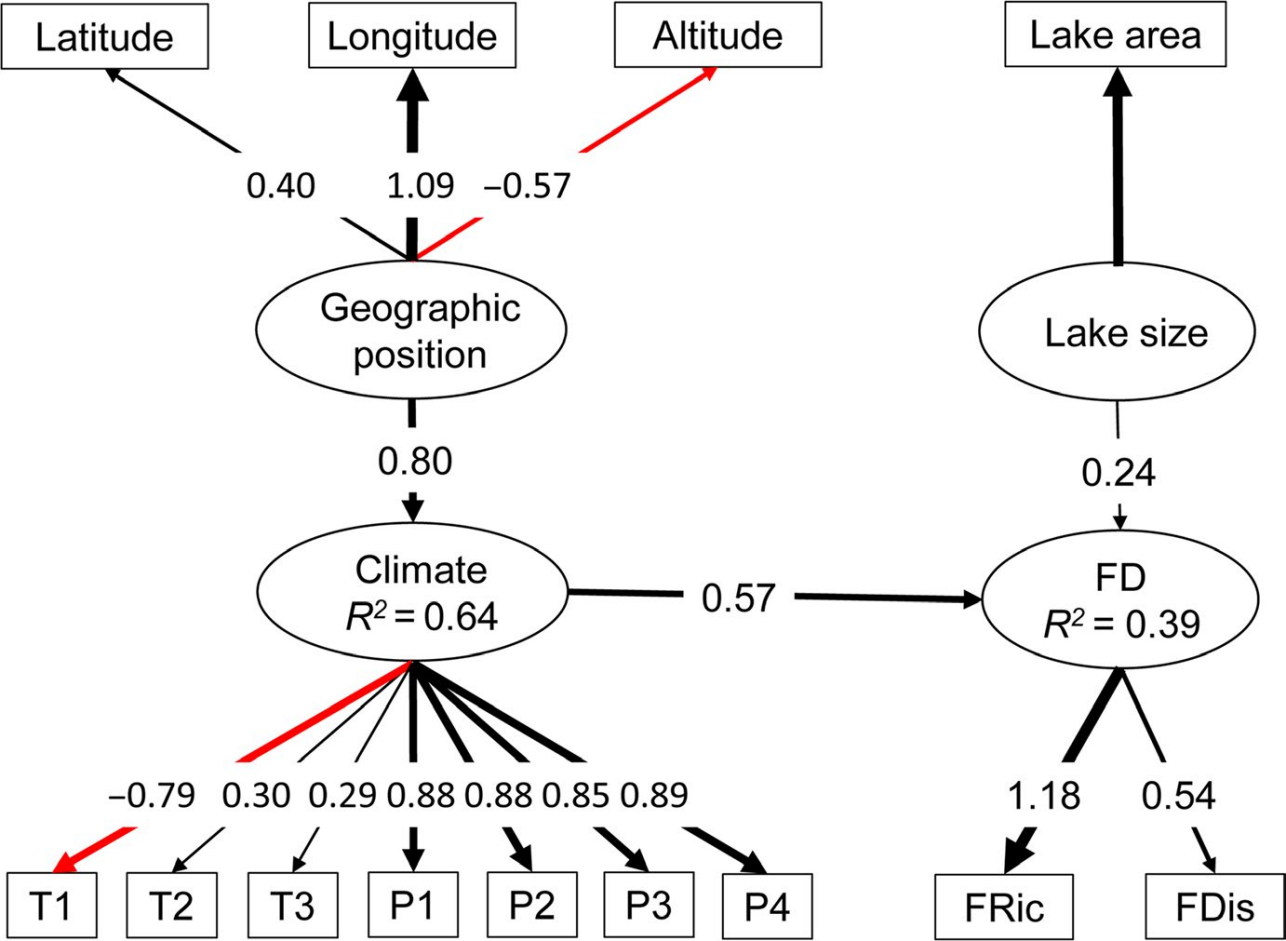
SEM model for waterbird functional diversity. Latent variables in ovals and measured variables in rectangles. The strength of the causality (standardized path coefficient for direct comparison) was indicated by the width of the link. Black lines indicate positive effects, while red lines mean negative impacts. T1: mean diurnal range, T2: mean temperature of the wettest quarter, T3: mean temperature of the warmest quarter, P1: annual total precipitation, P2: precipitation of the driest month, P3: precipitation of the warmest quarter, and P4: precipitation of the coldest quarter

Similar to the lake productivity model, the three latent factors (geographic position, climate, and lake size) were all included in the SEM, collectively explaining 39% of the variation in waterbird FD (Figure 5). In comparison with lake size, climate had higher effects on waterbird FD (path coefficient = 0.57 and 0.24 for climate and area, respectively, Figure 5). The effects of geographic position on waterbird distribution were indirect through climate with an effect of 0.46 (0.80 × 0.57 = 0.46). Based on the standardized model coefficients, climate had the strongest effects on waterbird FD, followed by geographic position and lake size (the coefficients are 0.57, 0.46, and 0.24 for climate, geographic position, and lake size, respectively, Figure 5).

Waterbird FD decreased with elevation (path coefficient = −0.57 × 0.80 × 0.57 =−0.26) and increased with both latitude and longitude, with longitude showing much stronger effect than latitude (path coefficient = 0.50 and 0.18, respectively, Figure 5). For the measured bioclimatic variables, similar to the productivity model, while all precipitation variables ad well as T2 and T3 had positive effects on waterbird FD, the impact of temperature diurnal range (T1) was negative. In addition, the four precipitation variables had comparable effects on FD (Figure 5).

### 3.4 Relationship between waterbird functional diversity and lake productivity

**FIGURE 6:**
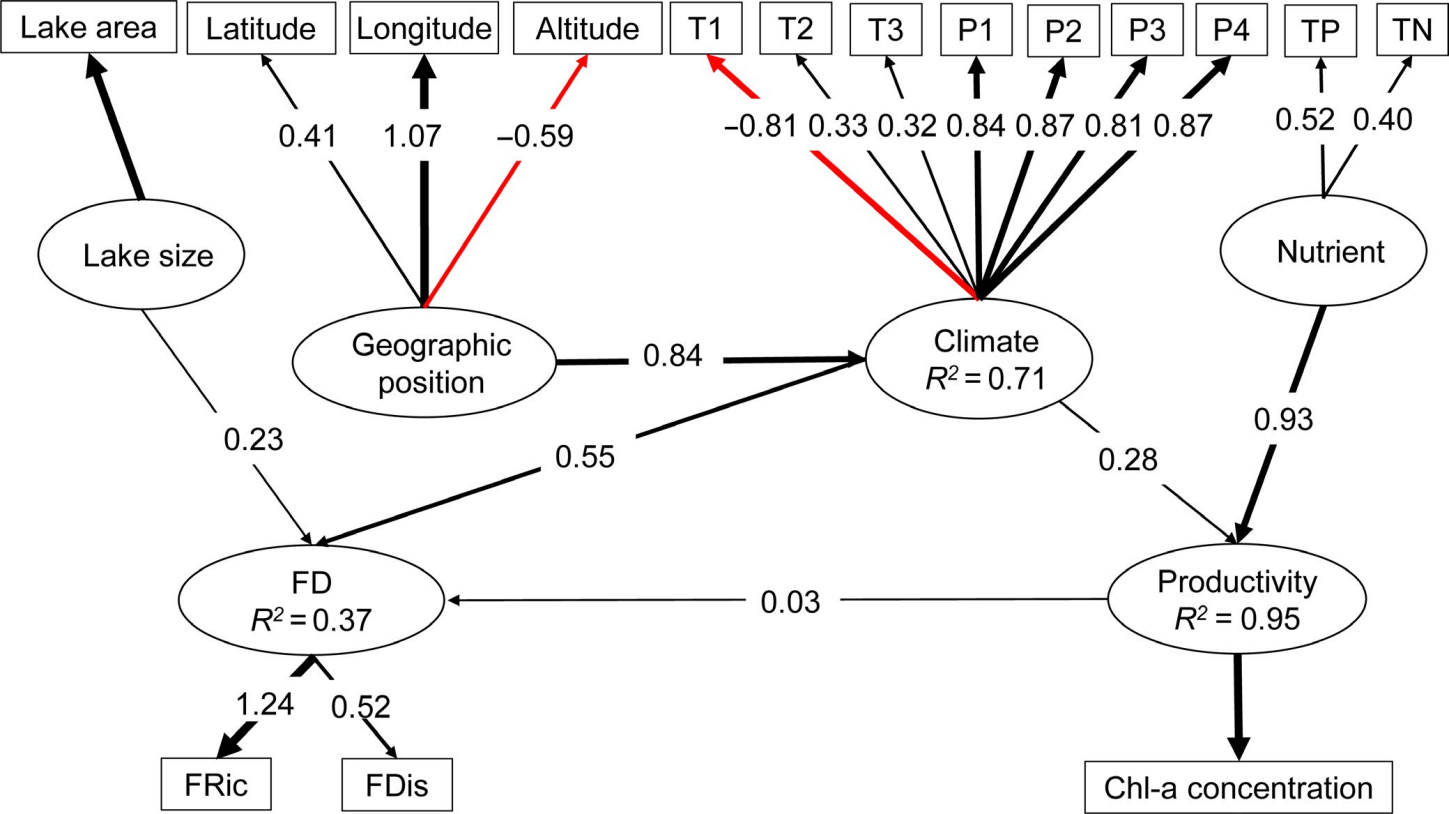
Integrated SEM model in which waterbird functional diversity is linked with lake productivity. Latent variables are in ovals and measured variables in rectangles. The strength of the causality (standardized path coefficient) was indicated by the width of the link. Black lines indicate positive effects, while red lines mean negative impacts. T1: mean diurnal range, T2: mean temperature of the wettest quarter, T3: mean temperature of the warmest quarter, P1: annual total precipitation, P2: precipitation of the driest month, P3: precipitation of the warmest quarter, and P4: precipitation of the coldest quarter

The final integrated *SEM* combined lake productivity and waterbird FD together and included an explicit pathway from productivity to waterbird FD (Figure 6). The model had reasonably adequate explanation power for the three key latent variables (*R^2^* for climate, waterbird FD, and lake productivity was 0.71, 0.37, and 0.95, respectively. Figure 6). From the fitted path coefficients, climate had the greatest effect on waterbird FD (0.55), which were similar to that of the waterbird FD model (Figure 5). The effects of geographic position (0.47) were relatively strong, while the effects of other variables, including lake size (0.23), lake productivity (0.03), and nutrient (0.03, indirectly via its effect on lake productivity), were relatively weak.

## 4 DISCUSSION

In this study, we applied integrated modeling procedures (SEM) to lake productivity and waterbird FD data collected from lakes and reservoirs in the arid and semi‐arid northern China covering over 5 million km^2^ with the aim to explore the mechanisms underlying geographic gradients in inland aquatic systems. Both lake productivity and waterbird FD displayed strong geographical variations across northern China (Figure 3). We found that the geographic position exerted effects on lake productivity and waterbird FD in a similar way, that is, through its influences on climatic conditions, which was defined by seven bioclimatic variables in this study. This causality from geographic position to climatic conditions was significant and consistent in all three SEMs. Specifically, our analyses showed an unambiguous decreasing altitudinal gradient for both lake productivity and waterbird FD (Herzog, Kessler, & Bach, 2005; Rahbek, 1995). An increasing gradient with location coordinates was also obvious; and the effects of latitude (through its effects on climate) on both lake productivity and waterbird FD were relatively weaker than those of longitude. Moreover, the ‘‘latitudinal gradient,’’ which predicts species diversity decreases when moving away from the equator toward northern latitudes (Jetz, Thomas, Joy, Hartmann, & Mooers, 2012), was not supported. Instead, we found evidence of a reverse trend in our analyses. The opposite latitudinal gradient in this study should be treated with cautions as the study focused on the temperate zone that have a rather narrow latitudinal range and strong negative effects of elevation might obscure the real latitudinal pattern. Nevertheless, our analysis results supported some hypotheses underlying the geographic gradients by Field et al. (2009): (a) climate and (b) productivity. More importantly, our results revealed that both geographical and environmental factors regulate the observed biodiversity and productivity patterns (Macneil et al., 2009).

### 4.1 Drivers of geographic and altitudinal patterns of lake productivity

The lake productivity model had relatively high performance in explaining the observed geographic and altitudinal gradients (*R^2^* for separated and integrated models was 0.93 and 0.95, respectively, Figures 4 and 6), which showing both direct and indirect (through its effect on the climatic conditions) effects on lake productivity. Structural equation modeling (Figures 4 and 6) showed that lake productivity was positively related with water TN and TP concentration. Compared with other factors, nutrient had the strongest effects on lake productivity in our study. The close relationship between nutrient, lake chlorophyll *a* concentration, and lake productivity (Jeppesen et al., 2005; Smith, 1979) was expected because nitrogen and phosphorus are important limiting nutrients in freshwater ecosystems (Elser et al., 2007; Schindler, 1974). Lake productivity depends on the supply of nutrients, especially phosphorus (Wetzel, 2001). According to previous studies, phosphorus loading alone could explain 79%–95% of the variances in lake chlorophyll *a* concentration (Schindler, 1978).

Climatic conditions, measured mainly by temperature and precipitation variables including mean temperature of the wettest quarter, mean temperature of the warmest quarter, annual total precipitation, precipitation of driest month, and precipitation of the warmest quarter also had positive effects on lake productivity, except for mean diurnal range which showed the opposite trend (Figures 4 and 6). Climatic variation has been found to influence the magnitude of chlorophyll *a* concentration (O'Reilly, Alin, Plisnier, Cohen, & Mckee, 2003). Lake productivity increased with air temperature, which is the function of solar energy input (Danilov & Ekelund, 2001). Warner and Lesht (2015) reported that air temperature and precipitation were identified as important predictors, which had positive effects on chlorophyll *a*. Our results are consistent with those studies, although the mechanisms are not clear. One possibility is that higher air temperature reduces ice cover, which facilitates wind‐induced mixing and nutrient resuspension (Nicholls, 1998; Schwab, Eadie, Assel, & Roebber, 2009), and then having an impact on lake productivity (Warner & Lesht, 2015); inversely, ice cover shading has an effect on the benthic habitat of lakes, which reduces primary production (Toro, Granados, Robles, & Montes, 2006). In addition, air temperature could impact lake productivity through increasing water discharge or hydrograph fluctuations. For example, Toro et al. (2006) revealed that due to the increased air temperature and day/night temperature difference (mean diurnal range), water discharge from the snowpack to the high‐mountain lakes and hydrograph fluctuations increased, which in turn impacted lake environment including productivity. Increases in the form of rain would also cause increased runoff, which could bring more nutrients to the lakes from nonmonitored or diffuse nonpoint sources, thus increase the concentration of chlorophyll *a* (Dillon & Rigler, 1974). Rather, in our study, besides mean diurnal range, it was mean temperature of the wettest quarter, mean temperature of the warmest quarter showed significant effects on lake productivity. For which, it may due to the sampling time (i.e., we monitored lake chl‐*a* in summer). Our results indicate that further research will be required to understand more completely the underlining mechanisms by which climate influences lake productivity.

### 4.2 Drivers of geographic and altitudinal patterns of waterbird functional diversity

The waterbird FD SEMs achieved relatively sufficient performance in explaining the spatial variations in waterbird FD (*R^2^* for both separated and integrated models was 0.39 and 0.37, respectively, Figures 5 and 6). Geographic position, defined by latitude, longitude, and altitude, had dominating effect on waterbird FD through its influence on climate. Climate is typically a strong descriptor of broadscale richness patterns (Hawkins et al., 2003), and the theory that climate's control of energy drives the global richness gradient has generated an extensive literature quantifying the relationship between species richness and climatic variables (Whittaker et al., 2007). Our results demonstrated that waterbird FD increased with temperature, giving empirical affirmation to the species–energy hypothesis in that species diversity increases with environmental temperature (Allen, Brown, & Gillooly, 2002). Temperature is one of major determinants of latitudinal and altitudinal gradients in animal diversity (Allen et al., 2002; Rohde, 1992), which may be explained by energy hypothesis, although the underlying mechanism remains unknown (Hawkins et al., 2003). The model results also showed that waterbird FD increased significantly with precipitation. Furthermore, the modeled path coefficients indicated that precipitation was more important than that of temperature in our system. Precipitation is one of the resource‐based estimates of available energy, especially in arid and semi‐arid ecosystems (Brown & Davidson, 1977), where biodiversity patterns are strongly related to precipitation amount (Waide et al., 1999). This is particularly true for waterbirds, whose distribution is generally determined by rainfall (Wen et al., 2016) through changing habitat availability, like water depth, habitat area, and habitat diversity (Canepuccia, Isacch, Gagliardini, Escalante, & Iribarne, 2007). Water depth is paramount in determining whether or not habitat is available (Bolduc & Afton, 2008). As most of the lakes included in our study are located in the arid and semi‐arid region of China, precipitation is critical to maintain enough habitat area for waterbirds.

Lake size, as expected, had a positive effect on waterbird FD. This pattern resembles the common species–area relationship observed in many ecosystems (Arrhenius, 1921; Guadagnin, Maltchik, & Fonseca, 2009; Keil, Storch, & Jetz, 2015; MacArthur & Wilson, 1967; Nogues‐Bravo & Araujo, 2006; Rosenzweig, 1995). According to the theory of island biogeography (MacArthur & Wilson, 1967), large and more diverse ecosystems are likely to harbor more species due to higher immigration rates and lower extinction rates. Indeed, lakes in arid and semi‐arid zones can be regarded as aquatic islands in a terrestrial world, offering an explanation for the positive species–area relation in our analysis. This finding is consistent with other studies (Froneman, Mangnall, Little, & Crowe, 2001; Guadagnin et al., 2009; Suter, 1994), which all reported a positive species–area relationship.

### 4.3 Relationship between waterbird functional diversity and lake productivity

Many studies revealed that productivity affects diversity (Carpenter et al., 1987; Dodson et al., 2000; Mittelbach et al., 2001), especially for plants (Chase & Leibold, 2002). Nonetheless, no general consensus concerning the form of the pattern has emerged based on theoretical considerations or empirical findings (Waide et al., 1999). Positive, negative, and hump‐shaped patterns were common at most spatial scales and no one pattern predominated (Mittelbach et al., 2001). For avian species, particularly waterbirds, there are only a few studies presented the relationship between diversity and productivity (Hawkins et al., 2003; Hurlbert, 2004). Results of our integrated SEM gave evidence to support the causality from lake productivity to waterbird FD albeit the relationship was weak in comparison with other factors. The weak causality is also reflected in that the explained variations in waterbird FD were not improved by the inclusion of lake productivity in the SEM. In this study, the majority of waterbirds forage on the riparian zone of lakes (mean trait value of foraging at ground was greater than 55% for all lakes, Figure S2) and have plants as their major diets (Figure S3), suggesting that the composition of waterbird communities could be more affected by riparian areas than by lake productivity per se. Because lake productivity was measured solely by water column chlorophyll *a* in this study, this weak causality is expected. A broader definition of lake productivity, that is, incorporating factors operating outside of water column (e.g., riparian meadows and mudflats), would result in a closer relationship and higher model performance.

### 4.4 Conclusions and caveats

A major contribution of this study is that our findings reveal the key environmental drivers of large‐scale patterns in lake productivity and waterbird FD in the arid and semi‐arid region of northern China using advanced statistical techniques (i.e., SEM). This approach showed that the observed geographical and altitudinal gradients in lake productivity and waterbird FD (Figure 3) can be partly explicated by the gradients in climatic conditions, which is in term significantly related to the geographic position of the lakes on the earth surface. As the relationship between productivity and waterbird FD in arid and semi‐arid ecosystems has not been addressed, our study could contribute to the mechanistic explanations underlying the observed broadscale biodiversity gradients in arid and semi‐arid region. However, site‐specific factors, such as lake size (for waterbirds) and nutrients (for productivity), impose their effects independently (Figures 4, 5, 6), and their effects could be more important than climatic variables (e.g., for lake productivity). These results, although supporting some primary macroecological biodiversity theories such as species–energy and species–resource hypothesis, could not lead to a mechanism that unifies these theories (Mcgill, 2010), exemplifying one key limitation of statistical analyses: statistical relationships do not necessarily reveal the underlying mechanisms regulating waterbird biodiversity (Stomp et al., 2011). For example, the SEM indicates that altitude has a strong negative effect (indirectly through climate and lake productivity, Figure 6) on waterbird FD. However, these causal paths could be driven by other environmental variables that covary with altitude but were not measured in our study. For instance, seasonal variation in environmental conditions increases at higher elevation, which could reduce species diversity by excluding sensitive species with a narrow tolerance range (Currie et al., 2004). Nevertheless, through articulating the dominant processes, our results could contribute to future studies seeking mechanistic explanations underlying the observed macroecological phenomena.

## CONFLICT OF INTEREST

None declared.

## AUTHOR CONTRIBUTION

**Yamian Zhang:** Conceptualization (lead); Formal analysis (lead); Investigation (lead); Methodology (equal); Writing‐original draft (lead); Writing‐review & editing (lead). **Wenzhuo Tan:** Formal analysis (supporting); Investigation (lead); Writing‐review & editing (supporting). **QIng Zeng:** Investigation (equal); Methodology (supporting); Writing‐review & editing (supporting). **Haitao Tian:** Investigation (equal); Writing‐review & editing (supporting). **Yifei Jia:** Investigation (equal); Writing‐review & editing (supporting). **Guangchun Lei:** Conceptualization (lead); Data curation (lead); Funding acquisition (lead); Methodology (supporting); Writing‐review & editing (lead). **Li Wen:** Conceptualization (lead); Data curation (lead); Formal analysis (lead); Methodology (lead); Writing‐review & editing (lead).

## Supporting information

Appendix S1

## Data Availability

The data used in this study have been archived through Dryad online data repository and are publically available at https://doi.org/10.5061/dryad.mpg4f4qwd.
