# Supplementary material for: Lake productivity and waterbird functional diversity across geographic and environmental gradients in temperate China
Source: Ecol Evol. 2020 Sep 23;10(20):11237–50. doi: 10.1002/ece3.6763 (PMC7593163; doi:10.1002/ece3.6763)

**Appendices**

**Table S1** Data of the 35 lakes and reservoirs used in this study.

| lake name | Species richness | FRic | FDis | Productivity (mg.m^-3^) | Latitude | Longitude | Lake area (ha) | Altitude (m) | TN (mg.L^-1^) | TP (mg.L-1) | Mean diurnal range (℃) | Mean temperature of wettest quarter (℃) | Mean temperature of warmest quareter (℃) | Annual precipitation (mm) | Precipitation of driest month (mm) | Precipitation of warmest quarter (mm) | Precipitation of coldest quarter (mm) |
| --- | --- | --- | --- | --- | --- | --- | --- | --- | --- | --- | --- | --- | --- | --- | --- | --- | --- |
| Wuliangsuhai | 96 | 0.0963 | 0.1538 | 11.36 | 40.93 | 108.83 | 29300.00 | 1017.42 | 3.65 | 0.18 | 13.17 | 19.95 | 21.91 | 230.14 | 1.00 | 153.65 | 5.00 |
| Hasuhai | 51 | 0.0555 | 0.1421 | 8.36 | 40.6 | 110.98 | 4161.54 | 1052.99 | 3.12 | 0.05 | 13.29 | 19.11 | 21.11 | 354.30 | 4.00 | 228.30 | 13.46 |
| Dalinuoer | 99 | 0.1047 | 0.1594 | 3.30 | 43.25 | 116.42 | 38536.07 | 1317.72 | 0.89 | 0.08 | 13.22 | 17.30 | 17.30 | 342.36 | 2.03 | 231.94 | 8.31 |
| Erdos | 72 | 0.0928 | 0.1628 | 10.13 | 36.71 | 109.31 | 1717.43 | 1192.04 | 15.28 | 0.24 | 12.50 | 19.30 | 20.70 | 502.00 | 3.00 | 279.00 | 11.00 |
| Chagannaoer | 72 | 0.0896 | 0.1605 | 72.92 | 43.43 | 114.92 | 3300.00 | 1048.06 | 3.19 | 0.14 | 13.60 | 19.75 | 19.75 | 253.00 | 2.00 | 174.00 | 6.00 |
| Wulagai | 103 | 0.0976 | 0.1529 | 4.62 | 46.06 | 119.57 | 22600.00 | 1003.27 | 3.73 | 0.13 | 13.76 | 16.69 | 16.69 | 409.25 | 3.72 | 282.08 | 11.72 |
| Juyanhai | 40 | 0.0642 | 0.1588 | 2.62 | 42.33 | 101.25 | 4000.00 | 917.29 | 0.48 | 0.21 | 14.90 | 24.50 | 24.50 | 35.00 | 0.00 | 26.00 | 0.00 |
| Daihai | 43 | 0.0639 | 0.1482 | 25.35 | 40.55 | 112.66 | 2420.00 | 1297.61 | 13.16 | 0.12 | 13.21 | 19.37 | 19.37 | 385.48 | 2.00 | 259.50 | 8.23 |
| Khorchin | 66 | 0.0824 | 0.1613 | 69.42 | 45.08 | 122.18 | 18047.04 | 169.37 | 15 | 0.27 | 12.20 | 22.11 | 22.11 | 404.55 | 1.00 | 299.35 | 5.00 |
| Hangjinnaoer | 55 | 0.0721 | 0.1477 | 18.32 | 40.5 | 108.96 | 1600.00 | 1059.96 | 0.88 | 0.25 | 12.95 | 19.97 | 21.92 | 259.87 | 1.00 | 170.02 | 5.72 |
| Naihai Wetland | 55 | 0.0736 | 0.1550 | 96.95 | 40.53 | 110.01 | 1308.25 | 1034.10 | 1.32 | 0.38 | 12.80 | 19.50 | 21.60 | 308.00 | 2.00 | 193.00 | 8.00 |
| Honggou reservoir | 4 | 0.0001 | 0.1132 | 14.60 | 38.84 | 105.67 | 160.00 | 55.32 | 4.73 | 0.2 | 13.38 | 19.76 | 21.49 | 202.97 | 1.84 | 118.40 | 5.84 |
| Sand Lake | 74 | 0.0796 | 0.1534 | 11.43 | 38.62 | 106.33 | 8602.23 | 1117.78 | 0.67 | 0.22 | 13.16 | 20.59 | 22.30 | 192.33 | 1.00 | 112.93 | 4.00 |
| Habahu | 45 | 0.0673 | 0.1555 | 10.07 | 37.83 | 107.28 | 240.00 | 1479.40 | 0.57 | 0.23 | 13.22 | 18.83 | 20.44 | 289.34 | 1.21 | 163.16 | 6.21 |
| Mingcuihu | 74 | 0.0837 | 0.1507 | 14.85 | 38.38 | 106.37 | 492.00 | 1140.53 | 0.6 | 0.22 | 13.10 | 20.40 | 22.10 | 203.00 | 1.00 | 118.00 | 4.00 |
| Yuehai | 37 | 0.0609 | 0.1521 | 39.34 | 38.57 | 106.21 | 1070.37 | 1120.97 | 0.83 | 0.27 | 13.10 | 20.60 | 22.27 | 192.83 | 1.00 | 112.83 | 4.00 |
| Xinghaihu | 24 | 0.0371 | 0.1458 | 55.21 | 38.99 | 106.41 | 3283.91 | 1179.46 | 1.12 | 0.27 | 13.60 | 20.96 | 22.72 | 177.00 | 1.00 | 106.00 | 4.00 |
| Tianhu | 24 | 0.0371 | 0.1458 | 6.65 | 38.02 | 105.74 | 591.00 | 1356.68 | 0.55 | 0.22 | 13.60 | 19.80 | 21.40 | 212.00 | 1.00 | 123.00 | 4.00 |
| Hongjiannao | 74 | 0.0829 | 0.1572 | 1.75 | 39.1 | 109.88 | 3330.00 | 1266.89 | 1.09 | 0.06 | 12.75 | 19.15 | 20.92 | 404.10 | 2.00 | 253.05 | 9.00 |
| Kusaihu | 9 | 0.0172 | 0.1514 | 0.41 | 35.73 | 92.83 | 32600.00 | 4554.99 | 0.78 | 0.11 | 14.20 | 5.42 | 5.42 | 201.11 | 1.00 | 139.64 | 3.06 |
| Goulucuohu | 9 | 0.0227 | 0.1605 | 0.49 | 34.6 | 92.47 | 2350.00 | 4762.46 | 0.67 | 0.12 | 15.00 | 5.80 | 5.80 | 269.00 | 0.00 | 191.00 | 4.00 |
| Zhuonaihu | 8 | 0.0124 | 0.1395 | 0.51 | 35.55 | 91.95 | 16300.00 | 4821.87 | 0.9 | 0.21 | 14.45 | 4.26 | 4.26 | 202.77 | 0.08 | 144.73 | 3.04 |
| Yanhu | 9 | 0.0187 | 0.1511 | 0.51 | 35.53 | 93.42 | 1120.00 | 4480.50 | 0.68 | 0.1 | 14.10 | 6.20 | 6.20 | 213.00 | 1.00 | 144.00 | 3.00 |
| Chaganhu | 94 | 0.0891 | 0.1569 | 40.00 | 45.26 | 124.29 | 51936.54 | 123.46 | 1.56 | 1.05 | 11.78 | 22.10 | 22.10 | 433.57 | 2.00 | 305.30 | 7.51 |
| Hengshuihu | 113 | 0.1002 | 0.1599 | 16.30 | 37.69 | 115.58 | 18787.00 | 22.49 | 1.12 | 0.21 | 11.34 | 26.41 | 26.41 | 454.96 | 4.00 | 331.03 | 14.56 |
| Cuihu | 56 | 0.0688 | 0.1554 | 15.00 | 40.08 | 116.17 | 166.00 | 157.30 | 1.76 | 0.12 | 11.50 | 25.50 | 25.50 | 669.00 | 4.00 | 533.00 | 13.00 |
| Alagou | 8 | 0.0080 | 0.1601 | 1.06 | 42.85 | 87.36 | 94.00 | 2893.38 | 1.62 | 0.06 | 12.40 | 16.80 | 16.80 | 196.00 | 1.00 | 113.00 | 5.00 |
| Bosten Lake | 58 | 0.0823 | 0.1605 | 8.98 | 41.67 | 86.9 | 94529.19 | 1525.86 | 0.7 | 0.11 | 13.60 | 22.23 | 22.23 | 81.22 | 0.17 | 51.12 | 1.75 |
| Heshilike | 17 | 0.0172 | 0.1343 | 51.07 | 41.75 | 85.69 | 525.00 | 890.18 | 1 | 0.08 | 12.60 | 25.20 | 25.20 | 56.00 | 1.00 | 34.00 | 4.00 |
| Kongquehe | 17 | 0.0172 | 0.1343 | 39.62 | 41.25 | 86.5 | 79.25 | 876.60 | 12.19 | 0.5 | 13.80 | 25.60 | 25.60 | 45.00 | 0.00 | 27.00 | 2.00 |
| Kenadaliya | 17 | 0.0172 | 0.1343 | 5.76 | 38.61 | 86.29 | 69.90 | 1059.16 | 1.14 | 0.05 | 16.30 | 25.10 | 25.10 | 21.00 | 0.00 | 18.00 | 0.00 |
| Taitemahu Lake | 31 | 0.0517 | 0.1518 | 6.65 | 39.48 | 88.29 | 26930.42 | 799.27 | 0.87 | 0.03 | 16.40 | 26.35 | 26.35 | 23.86 | 0.00 | 16.07 | 1.79 |
| Ayakekumuhu Lake | 21 | 0.0466 | 0.1582 | 2.74 | 37.52 | 89.79 | 98800.00 | 4057.34 | 1.56 | 0.08 | 14.64 | 7.72 | 7.72 | 91.08 | 0.00 | 68.16 | 1.01 |
| Miyun Reservior | 113 | 0.1020 | 0.1611 | 3.55 | 40.48 | 116.83 | 18800.00 | 242.67 | 1.29 | 0.03 | 12.30 | 24.01 | 24.01 | 531.88 | 3.00 | 403.55 | 10.06 |
| Yehahu Lake | 105 | 0.0895 | 0.1522 | 5.79 | 40.46 | 115.84 | 441.00 | 777.39 | 0.82 | 0.08 | 12.20 | 23.40 | 23.40 | 440.00 | 3.00 | 333.00 | 10.00 |

**Tabl S2** Biometric trait data of for all 148 waterbird species recorded in this study.

| Waterbirds | Generation | Clutch | Incubation | Migratory | Size | Mass | DietV | DietInv | DietScav | DietP | Water | Riparian | Ground | Pelagic | Wingspan | Range |
| --- | --- | --- | --- | --- | --- | --- | --- | --- | --- | --- | --- | --- | --- | --- | --- | --- |
| *Actitis_hypoleucos* | 6.8 | 4 | 21.5 | A | 20 | 48 | 40 | 50 | 0 | 10 | 0 | 50 | 50 | 0 | 39.5 | 7.67 |
| *Aix_galericulata* | 7.4 | 10.4 | 30.5 | A | 46 | 567.04 | 10 | 20 | 0 | 70 | 0 | 100 | 0 | 0 | 71 | 6.76 |
| *Amaurornis_phoenicurus* | 3.7 | 6 | 20 | A | 30.5 | 180 | 20 | 40 | 0 | 40 | 0 | 20 | 60 | 0 | 49 | 7.59 |
| *Anas_acuta* | 6.8 | 7.9 | 23 | A | 58 | 944.62 | 10 | 20 | 0 | 70 | 0 | 70 | 30 | 0 | 87.5 | 7.62 |
| *Anas_clypeata* | 6.5 | 9.9 | 22 | A | 49.5 | 612.56 | 0 | 60 | 0 | 40 | 20 | 80 | 0 | 0 | 77.5 | 7.60 |
| *Anas_crecca* | 6.3 | 9.4 | 22 | A | 36 | 341.89 | 0 | 30 | 0 | 70 | 0 | 100 | 0 | 0 | 61 | 7.68 |
| *Anas_falcata* | 6.6 | 7.3 | 25 | A | 46 | 645.83 | 0 | 10 | 0 | 90 | 0 | 70 | 30 | 0 | 80 | 6.93 |
| *Anas_formosa* | 6.6 | 7.3 | 24.5 | A | 41 | 433.98 | 0 | 10 | 0 | 90 | 0 | 70 | 30 | 0 | 70 | 6.69 |
| *Anas_penelope* | 6.4 | 8.5 | 24.5 | A | 48 | 770.03 | 0 | 0 | 0 | 100 | 0 | 50 | 50 | 0 |  | 7.54 |
| *Anas_platyrhynchos* | 6.6 | 10.8 | 27 | A | 58.75 | 843.42 | 20 | 40 | 0 | 40 | 20 | 60 | 20 | 0 |  | 7.82 |
| *Anas_poecilorhyncha* | 6.6 | 7.9 | 26 | A | 60.5 | 999.96 | 0 | 10 | 0 | 90 | 0 | 50 | 50 | 0 |  | 7.00 |
| *Anas_querquedula* | 6.5 | 8.5 | 22 | A | 39 | 325.6 | 20 | 40 | 0 | 40 | 100 | 0 | 0 | 0 | 63.5 | 7.51 |
| *Anas_strepera* | 6.6 | 9.8 | 24 | A | 46 | 915.58 | 0 | 0 | 0 | 100 | 0 | 80 | 20 | 0 | 89.5 | 7.86 |
| *Anser_albifrons* | 11.3 | 5.5 | 25 | A | 75.5 | 2506.39 | 0 | 0 | 0 | 100 | 0 | 20 | 80 | 0 | 147.5 | 7.28 |
| *Anser_anser* | 11.6 | 4.9 | 27.5 | A | 82.5 | 3302.41 | 0 | 0 | 0 | 100 | 0 | 20 | 80 | 0 | 163.5 | 7.49 |
| *Anser_cygnoides* | 11.4 | 5.5 | 28 | A | 87 | 3511.94 | 0 | 0 | 0 | 100 | 0 | 20 | 80 | 0 | 175 | 6.58 |
| *Anser_erythropus* | 11.4 | 4.9 | 26.5 | A | 59.5 | 1755.5 | 0 | 0 | 0 | 100 | 0 | 20 | 80 | 0 | 125 | 6.85 |
| *Anser_fabalis* | 11.4 | 4.9 | 27 | A | 77.5 | 2754.73 | 0 | 0 | 0 | 100 | 0 | 20 | 80 | 0 | 158.5 | 7.31 |
| *Anser_indicus* | 11.4 | 4.9 | 28.5 | A | 73.5 | 2212.55 | 0 | 0 | 0 | 100 | 0 | 20 | 80 | 0 | 150 | 6.75 |
| *Ardea_cinerea* | 10.3 | 3.9 | 25.5 | A | 94 | 1443 | 80 | 20 | 0 | 0 | 0 | 70 | 30 | 0 | 185 | 8.13 |
| *Ardea_purpurea* | 10.5 | 4 | 27.5 | A | 84 | 1064.48 | 70 | 30 | 0 | 0 | 0 | 70 | 30 | 0 | 135 | 8.12 |
| *Ardeola_bacchus* | 6.7 | 4 | 20 | A | 47 | 304.89 | 30 | 70 | 0 | 0 | 20 | 70 | 10 | 0 | 84.5 | 6.96 |
| *Arenaria_interpres* | 7.3 | 4 | 23 | A | 23.5 | 135.98 | 0 | 80 | 0 | 20 | 0 | 0 | 100 | 0 | 53.5 | 5.25 |
| *Aythya_baeri* | 7.6 | 7.7 | 27 | A | 44 | 681.23 | 20 | 20 | 0 | 60 | 100 | 0 | 0 | 0 | 70 | 6.36 |
| *Aythya_ferina* | 7.6 | 8.9 | 52.5 | A | 45.5 | 822.99 | 20 | 10 | 0 | 70 | 50 | 50 | 0 | 0 | 77 | 5.74 |
| *Aythya_fuligula* | 7.3 | 9.4 | 25.5 | A | 43.5 | 701.17 | 0 | 50 | 10 | 40 | 60 | 40 | 0 | 0 | 68.5 | 7.54 |
| *Aythya_marila* | 8.2 | 9.4 | 27 | A | 45.5 | 1005.37 | 0 | 80 | 0 | 20 | 80 | 20 | 0 | 0 | 78 | 7.67 |
| *Aythya_nyroca* | 7.6 | 8.9 | 26.5 | A | 40 | 574 | 20 | 10 | 0 | 70 | 50 | 50 | 0 | 0 | 65 | 7.40 |
| *Botaurus_stellaris* | 5.5 | 4.6 | 25.5 | A | 72 | 1319.45 | 90 | 10 | 0 | 0 | 0 | 50 | 50 | 0 | 130 | 7.94 |
| *Bubulcus_ibis* | 8.1 | 3.9 | 23.5 | A | 51 | 365.95 | 30 | 60 | 10 | 0 | 0 | 30 | 70 | 0 | 92 | 8.60 |
| *Bucephala_clangula* | 8 | 9.4 | 30 | A | 46 | 918.56 | 10 | 70 | 0 | 20 | 80 | 20 | 0 | 0 | 72.5 | 7.67 |
| *Butorides_virescens* | 5.1 | 3.9 | 19 | A | 41.5 | 201.5 | 70 | 30 | 0 | 0 | 0 | 80 | 20 | 0 | 52 | 8.48 |
| *Calidris_acuminata* | 7.4 | 4 |  | A | 19.5 | 66.08 | 0 | 80 | 0 | 20 | 0 | 20 | 80 | 0 | 39.5 | 5.82 |
| *Calidris_alpina* | 8.1 | 4 | 22 | A | 19 | 51.89 | 0 | 80 | 0 | 20 | 0 | 20 | 80 | 0 | 36.5 | 6.60 |
| *Calidris_canutus* | 6.8 | 3.5 | 21.5 | A | 24 | 141.87 | 10 | 80 | 0 | 10 | 0 | 0 | 100 | 0 | 49.5 | 7.25 |
| *Calidris_ferruginea* | 7.6 | 3.8 | 19.5 | A | 20.5 | 58.08 | 0 | 80 | 0 | 20 | 0 | 50 | 50 | 0 | 42 | 6.48 |
| *Calidris_melanotos* | 7.4 | 4 | 22 | A | 21 | 79.73 | 0 | 80 | 0 | 20 | 0 | 20 | 80 | 0 | 41 | 7.16 |
| *Calidris_minuta* | 6.8 | 4 | 20.5 | A | 13 | 21.1 | 0 | 70 | 0 | 30 | 0 | 50 | 50 | 0 | 29.5 | 6.68 |
| *Calidris_ruficollis* | 7.5 | 4 | 22 | A | 14.5 | 26.79 | 0 | 60 | 0 | 40 | 0 | 50 | 50 | 0 | 31 | 6.53 |
| *Calidris_subminuta* | 7.4 | 4 |  | A | 14.5 | 30.2 | 0 | 70 | 0 | 30 | 0 | 50 | 50 | 0 | 30.5 | 7.04 |
| *Calidris_temminckii* | 6.5 | 4 | 21.5 | A | 14 | 23 | 0 | 90 | 0 | 10 | 0 | 20 | 80 | 0 | 35.5 | 6.99 |
| *Calidris_tenuirostris* | 7.4 | 4 | 21 | A | 27 | 192 | 0 | 50 | 0 | 50 | 0 | 0 | 80 | 0 | 61 | 5.52 |
| *Casmerodius_albus* | 9.1 | 3.9 | 26 | A | 92 | 871.33 | 60 | 40 | 0 | 0 | 0 | 50 | 50 | 0 | 155 | 8.57 |
| *Charadrius_alexandrinus* | 5 | 3 | 26 | A | 15 | 42.3 | 0 | 100 | 0 | 0 | 0 | 40 | 60 | 0 | 43.5 | 7.85 |
| *Charadrius_asiaticus* | 5.8 | 3 |  | A | 19 | 77.09 | 0 | 80 | 0 | 20 | 0 | 0 | 100 | 0 | 58 | 6.49 |
| *Charadrius_dubius* | 5 | 4 | 25 | A | 15.5 | 38.7 | 0 | 100 | 0 | 0 | 0 | 20 | 80 | 0 | 45 | 7.75 |
| *Charadrius_hiaticula* | 5.1 | 3.5 | 24 | A | 19 | 64.04 | 0 | 100 | 0 | 0 | 0 | 0 | 100 | 0 | 52.5 | 7.61 |
| *Charadrius_leschenaultii* | 5.8 | 3 | 24 | A | 22.5 | 74.8 | 10 | 90 | 0 | 0 | 0 | 0 | 100 | 0 | 52 | 6.98 |
| *Charadrius_mongolus* | 5.2 | 3 | 23 | A | 19.5 | 64 | 0 | 100 | 0 | 0 | 0 | 40 | 60 | 0 | 51.5 | 7.67 |
| *Charadrius_veredus* | 5.2 | 3 |  | A | 22 | 95 | 0 | 80 | 0 | 20 | 0 | 0 | 100 | 0 | 49.5 | 6.26 |
| *Chlidonias_hybrida* | 9.9 | 2.5 | 19 | A | 26 | 83.67 | 60 | 40 | 0 | 0 | 20 | 50 | 0 | 0 | 67 | 8.14 |
| *Chlidonias_leucopterus* | 9.9 | 2.5 | 20 | A | 25 | 54.2 | 20 | 80 | 0 | 0 | 0 | 30 | 30 | 0 | 62.5 | 7.43 |
| *Chlidonias_niger* | 8.8 | 2.5 | 21.5 | A | 25.5 | 65.3 | 60 | 40 | 0 | 0 | 10 | 90 | 0 | 0 | 61 | 7.56 |
| *Ciconia_boyciana* | 16.1 | 3.5 | 33 | A | 112.5 | 4847.74 | 90 | 10 | 0 | 0 | 0 | 80 | 20 | 0 | 197.5 | 5.97 |
| *Ciconia_nigra* | 15.9 | 3.5 | 35 | A | 97.5 | 2926 | 90 | 10 | 0 | 0 | 0 | 100 | 0 | 0 | 149.5 | 7.40 |
| *Clangula_hyemalis* | 9 | 6.3 | 26.5 | A | 48.75 | 871 | 0 | 80 | 0 | 20 | 100 | 0 | 0 | 0 | 76 | 7.78 |
| *Cygnus_columbianus* | 12.7 | 3.9 | 31.5 | A | 135 | 6377.96 | 0 | 0 | 0 | 100 | 0 | 50 | 50 | 0 | 196 | 7.37 |
| *Cygnus_cygnus* | 12.3 | 4.5 | 35 | A | 152.5 | 9349.99 | 0 | 0 | 0 | 100 | 0 | 80 | 20 | 0 | 224 | 7.47 |
| *Cygnus_olor* | 12.2 | 5.9 | 38 | A | 142.5 | 10682.04 | 10 | 10 | 0 | 80 | 0 | 100 | 0 | 0 | 220 | 7.35 |
| *Egretta_eulophotes* | 6.6 | 3.2 | 23.5 | A | 66.5 | 461.83 | 40 | 60 | 0 | 0 | 0 | 100 | 0 | 0 | 99 | 6.09 |
| *Egretta_garzetta* | 6.6 | 3.5 | 23 | A | 60 | 312 | 40 | 60 | 0 | 0 | 0 | 70 | 30 | 0 | 95 | 8.18 |
| *Eudromias_morinellus* | 6.9 | 3 | 25 | A | 21 | 108.16 | 0 | 70 | 0 | 30 | 0 | 0 | 80 | 0 | 60.5 | 7.68 |
| *Fulica_atra* | 7 | 7.7 | 23.5 | A | 37.5 | 717.12 | 30 | 10 | 0 | 60 | 50 | 50 | 0 | 0 | 75 | 8.18 |
| *Gallicrex_cinerea* | 4.4 | 4.2 | 24 | A | 39.25 | 390.39 | 0 | 30 | 0 | 70 | 0 | 20 | 60 | 0 | 77 | 7.50 |
| *Gallinago_gallinago* | 4.8 | 4 | 19 | A | 26 | 112.94 | 0 | 80 | 0 | 20 | 0 | 20 | 80 | 0 | 45.5 | 7.33 |
| *Gallinago_megala* | 4.8 | 4 |  | A | 28 | 121 | 0 | 80 | 0 | 20 | 0 | 0 | 100 | 0 | 44 | 6.72 |
| *Gallinago_solitaria* | 4.8 | 4 |  | A | 30 | 140.11 | 0 | 100 | 0 | 0 | 0 | 0 | 100 | 0 | 53.5 | 7.01 |
| *Gallinago_stenura* | 4.8 | 4 | 20 | A | 26 | 113 | 0 | 80 | 0 | 20 | 0 | 0 | 100 | 0 | 45.5 | 7.11 |
| *Gallinula_chloropus* | 5.9 | 5.8 | 19.5 | A | 34 | 339.63 | 20 | 20 | 0 | 60 | 0 | 20 | 60 | 0 | 52.5 | 8.16 |
| *Gelochelidon_nilotica* | 11.7 | 2.5 |  | A | 38 | 218.25 | 50 | 50 | 0 | 0 | 0 | 30 | 30 | 1 | 94 | 8.21 |
| *Glareola_maldivarum* | 7.3 | 2.4 |  | A | 24 | 75.2 | 0 | 100 | 0 | 0 | 0 | 0 | 33 | 0 | 61 | 7.40 |
| *Grus_grus* | 13.4 | 2 | 29.5 | A | 107.5 | 5499.99 | 20 | 10 | 0 | 70 | 0 | 50 | 50 | 0 | 190 | 7.41 |
| *Grus_japonensis* | 12.3 | 2 | 31.5 | A | 145 | 8785.99 | 40 | 20 | 0 | 40 | 0 | 50 | 50 | 0 | 235 | 6.32 |
| *Grus_leucogeranus* | 13 | 2 | 28 | A | 140 | 5913.44 | 30 | 10 | 0 | 60 | 0 | 50 | 50 | 0 | 220 | 6.36 |
| *Grus_monacha* | 12 | 2 | 28.5 | A | 95.5 | 3729.9 | 20 | 10 | 0 | 70 | 0 | 50 | 50 | 0 | 170 | 6.42 |
| *Grus_nigricollis* | 13 | 2 | 32 | B | 115 | 5999.99 | 40 | 20 | 0 | 40 | 0 | 50 | 50 | 0 | 190 | 6.25 |
| *Grus_vipio* | 13 | 2 | 30 | A | 125 | 4662.99 | 10 | 20 | 0 | 70 | 0 | 50 | 50 | 0 | 205 | 6.15 |
| *Grus_virgo* | 11.2 | 2 | 25 | A | 95 | 1500 | 10 | 20 | 0 | 70 | 0 | 50 | 50 | 0 | 160 | 7.09 |
| *Haematopus_ostralegus* | 13.7 | 3 | 30 | A | 40 | 526 | 10 | 90 | 0 | 0 | 0 | 40 | 60 | 0 | 81 | 7.56 |
| *Heteroscelus_incanus* | 5.7 | 4 | 24 | A | 27.5 | 108.24 | 20 | 80 | 0 | 0 | 0 | 50 | 50 | 0 | 60 | 6.39 |
| *Himantopus_himantopus* | 7.3 | 3.2 | 25.5 | A | 37.5 | 176.82 | 0 | 100 | 0 | 0 | 0 | 100 | 0 | 0 | 75 | 8.56 |
| *Hydrophasianus_chirurgus* | 4.8 | 4 |  | A | 48.5 | 160.03 | 0 | 60 | 0 | 40 | 0 | 60 | 40 | 0 |  | 7.29 |
| *Hydroprogne_caspia* | 12.2 | 2 | 26.5 | A | 50.5 | 655 | 90 | 0 | 10 | 0 | 80 | 20 | 0 | 1 | 133.5 | 8.43 |
| *Ibidorhyncha_struthersii* | 9.6 | 4 |  | B | 40 | 294 | 10 | 90 | 0 | 0 | 0 | 100 | 0 | 0 | 74 | 6.87 |
| *Ixobrychus_cinnamomeus* | 4.1 | 3.9 |  | A | 40.5 | 126.49 | 70 | 30 | 0 | 0 | 0 | 80 | 20 | 0 |  | 7.42 |
| *Ixobrychus_eurhythmus* | 4.1 | 4.2 | 17 | A | 37.5 | 139 | 70 | 30 | 0 | 0 | 0 | 30 | 70 | 0 | 53.5 | 6.94 |
| *Ixobrychus_minutus* | 4.1 | 4.2 | 18.5 | A | 32.5 | 99.49 | 30 | 70 | 0 | 0 | 0 | 50 | 50 | 0 | 49 | 7.85 |
| *Ixobrychus_sinensis* | 4.1 | 4.6 | 18.5 | A | 35 | 94.29 | 20 | 80 | 0 | 0 | 0 | 50 | 50 | 0 | 49 | 7.71 |
| *Larus_argentatus* | 13 | 2.5 | 29 | A | 61 | 1090.99 | 50 | 30 | 20 | 0 | 20 | 40 | 40 | 1 | 125 | 6.86 |
| *Larus_brunnicephalus* | 11.5 | 3 | 24 | A | 43 | 569.29 | 50 | 30 | 10 | 10 | 0 | 50 | 50 | 0 |  | 5.93 |
| *Larus_canus* | 9.8 | 3 | 25 | A | 43 | 412.53 | 40 | 40 | 10 | 10 | 20 | 40 | 40 | 1 | 108.25 | 6.42 |
| *Larus_crassirostris* | 11.5 | 2.5 | 24.5 | A | 47 | 561 | 40 | 30 | 30 | 0 | 0 | 100 | 0 | 1 | 127 | 6.69 |
| *Larus_ichthyaetus* | 12.4 | 2 | 25 | A | 66 | 1378.84 | 70 | 20 | 0 | 10 | 10 | 50 | 40 | 0 | 162.5 | 6.65 |
| *Larus_minutus* | 10.5 | 2.4 | 24 | A | 27.5 | 118 | 20 | 80 | 0 | 0 | 10 | 60 | 30 | 0 | 74 | 7.53 |
| *Larus_relictus* | 6 | 2.4 | 25 | A | 44 | 490.2 | 60 | 40 | 0 | 0 | 0 | 50 | 50 | 0 |  | 6.43 |
| *Larus_ridibundus* | 9.6 | 1.7 | 24 | A | 40 | 284 | 20 | 70 | 0 | 10 | 0 | 50 | 50 | 0 | 102 | 7.65 |
| *Larus_saundersi* | 11.5 | 3 |  | A | 30.5 | 198 | 40 | 60 | 0 | 0 | 0 | 50 | 50 | 0 | 89 | 5.73 |
| *Larus_schistisagus* | 11.5 | 3 | 29 | A | 61 | 1323 | 40 | 30 | 20 | 10 | 30 | 40 | 30 | 1 | 140 | 6.71 |
| *Limicola_falcinellus* | 4.8 | 4 | 21.5 | A | 17 | 37.1 | 0 | 80 | 0 | 20 | 0 | 20 | 80 | 0 | 35.5 | 5.30 |
| *Limnodromus_semipalmatus* | 5.8 | 2 | 22 | A | 34.5 | 212 | 40 | 60 | 0 | 0 | 0 | 50 | 50 | 0 | 58 | 6.76 |
| *Limosa_lapponica* | 8.9 | 4 | 20.5 | A | 39 | 291.65 | 0 | 80 | 0 | 20 | 0 | 50 | 50 | 0 | 75 | 6.96 |
| *Limosa_limosa* | 8.6 | 4 | 23 | A | 40 | 288.37 | 20 | 60 | 0 | 20 | 0 | 50 | 50 | 0 | 76 | 7.48 |
| *Lymnocryptes_minimus* | 5.4 | 4 | 22.5 | A | 18 | 50.07 | 0 | 80 | 0 | 20 | 0 | 20 | 80 | 0 | 38 | 7.03 |
| *Melanitta_fusca* | 7.5 | 7.9 | 27.5 | A | 54.5 | 1800.17 | 10 | 80 | 0 | 10 | 80 | 20 | 0 | 0 | 92.5 | 7.17 |
| *Mergellus_albellus* | 5.7 | 7.9 | 27 | A | 39.5 | 608.55 | 20 | 70 | 0 | 10 | 100 | 0 | 0 | 0 | 62 | 7.24 |
| *Mergus_merganser* | 7.3 | 9.8 | 31 | A | 65 | 1451.02 | 90 | 10 | 0 | 0 | 80 | 20 | 0 | 0 | 89.5 | 7.91 |
| *Mergus_serrator* | 7.3 | 8.9 | 31.5 | A | 55 | 1015.17 | 70 | 20 | 0 | 10 | 80 | 20 | 0 | 0 | 78 | 7.81 |
| *Mesophoyx_intermedia* | 5.6 | 3.5 | 21 | A | 64 | 458.83 | 80 | 20 | 0 | 0 | 0 | 80 | 20 | 0 | 110 | 7.48 |
| *Netta_rufina* | 7 | 8.9 | 27 | A | 55.5 | 1118 | 0 | 0 | 0 | 100 | 50 | 50 | 0 | 0 | 87 | 7.29 |
| *Numenius_arquata* | 5 | 4 | 28 | A | 55 | 802.99 | 0 | 40 | 0 | 60 | 0 | 20 | 80 | 0 | 90 | 7.32 |
| *Numenius_madagascariensis* | 10.1 | 4 | 16 | A | 59.5 | 792 | 0 | 90 | 0 | 10 | 0 | 20 | 80 | 0 | 103.5 | 6.75 |
| *Numenius_minutus* | 10.1 | 4 | 22.5 | A | 31 | 173 | 0 | 80 | 0 | 20 | 0 | 0 | 80 | 0 | 69.5 | 6.24 |
| *Numenius_phaeopus* | 9.1 | 4 | 25 | A | 43 | 364.57 | 0 | 60 | 0 | 40 | 0 | 20 | 60 | 0 | 82.5 | 7.49 |
| *Nycticorax_nycticorax* | 8.8 | 3.9 | 22.5 | A | 60.5 | 810 | 70 | 30 | 0 | 0 | 0 | 50 | 50 | 0 | 108.5 | 8.47 |
| *Pelecanus_crispus* | 11 | 2 | 32 | A | 170 | 9512.09 | 100 | 0 | 0 | 0 | 100 | 0 | 0 | 0 |  | 7.10 |
| *Pelecanus_philippensis* | 15.4 | 3.5 | 30 | A | 139.5 | 5010.98 | 100 | 0 | 0 | 0 | 100 | 0 | 0 | 0 |  | 6.95 |
| *Phalacrocorax_carbo* | 11.3 | 3.5 | 29 | A | 90 | 2528.97 | 90 | 10 | 0 | 0 | 100 | 0 | 0 | 0 | 145 | 8.58 |
| *Phalaropus_fulicarius* | 9.9 | 4 |  | A | 38.5 | 308 | 0 | 100 | 0 | 0 | 0 | 0 | 80 | 0 | 38.5 | 7.32 |
| *Phalaropus_lobatus* | 4.9 | 4 | 21.5 | A | 21 | 36.68 | 0 | 80 | 0 | 20 | 0 | 50 | 50 | 0 | 36.5 | 6.70 |
| *Philomachus_pugnax* | 4.9 | 4 | 19 | A | 18.5 | 35.5 | 0 | 80 | 0 | 20 | 0 | 50 | 50 | 0 | 50 | 7.59 |
| *Platalea_leucorodia* | 7.2 | 3.5 | 24.5 | A | 82.5 | 1228 | 20 | 70 | 0 | 10 | 0 | 100 | 0 | 0 | 125 | 7.78 |
| *Pluvialis_fulva* | 5.6 | 4 | 23.5 | A | 24.5 | 250 | 0 | 70 | 0 | 30 | 0 | 0 | 80 | 0 | 66 | 5.85 |
| *Pluvialis_squatarola* | 6 | 4 | 26.5 | A | 29 | 84.11 | 0 | 70 | 0 | 30 | 0 | 0 | 100 | 0 | 77 | 6.46 |
| *Podiceps_auritus* | 7.1 | 4.5 | 23.5 | A | 34.5 | 730.96 | 60 | 40 | 0 | 0 | 70 | 30 | 0 | 0 | 327.5 | 7.72 |
| *Podiceps_cristatus* | 7.1 | 3.9 | 28 | A | 53.5 | 575 | 70 | 30 | 0 | 0 | 70 | 30 | 0 | 0 |  | 8.26 |
| *Podiceps_grisegena* | 7.1 | 4.5 | 22.5 | A | 45 | 1646 | 50 | 50 | 0 | 0 | 70 | 30 | 0 | 0 |  | 7.70 |
| *Podiceps_nigricollis* | 7.1 | 3.5 | 22 | A | 31 | 320.69 | 20 | 80 | 0 | 0 | 60 | 40 | 0 | 0 |  | 8.16 |
| *Porzana_fusca* | 2.7 | 5.2 | 20 | A | 22 | 49.7 | 0 | 40 | 0 | 60 | 0 | 20 | 80 | 0 |  | 7.50 |
| *Porzana_pusilla* | 2.7 | 6.6 | 18 | A | 18 | 61.06 | 10 | 70 | 0 | 20 | 0 | 50 | 50 | 0 | 29.5 | 7.87 |
| *Rallus_aquaticus* | 4.6 | 8.1 | 22 | A | 26.5 | 162.11 | 40 | 20 | 0 | 40 | 10 | 40 | 40 | 0 | 41.5 | 7.52 |
| *Recurvirostra_avosetta* | 8.7 | 3.5 | 24 | A | 43.5 | 325 | 10 | 80 | 0 | 10 | 0 | 50 | 50 | 0 | 78.5 | 7.88 |
| *Rissa_tridactyla* | 12.9 | 2 | 26 | A | 39 | 706 | 50 | 30 | 0 | 20 | 100 | 0 | 0 | 1 | 94 | 5.75 |
| *Rostratula_benghalensis* | 8.6 | 3.2 | 18 | C | 25.5 | 76.6 | 0 | 60 | 0 | 40 | 0 | 50 | 50 | 0 | 52.5 | 7.96 |
| *Scolopax_rusticola* | 6.3 | 4 | 17.5 | A | 34 | 205 | 0 | 70 | 0 | 30 | 0 | 20 | 80 | 0 | 58 | 7.61 |
| *Sterna_albifrons* | 10.9 | 2.5 | 22.5 | A | 25 | 94.69 | 60 | 40 | 0 | 0 | 100 | 0 | 0 | 1 | 47 | 8.45 |
| *Sterna_hirundo* | 11.5 | 2 | 25 | A | 35.5 | 53.8 | 80 | 10 | 10 | 0 | 80 | 20 | 0 | 0 | 77.5 | 7.93 |
| *Tachybaptus_ruficollis* | 5.4 | 3.7 | 22.5 | A | 27 | 3450 | 20 | 80 | 0 | 0 | 70 | 30 | 0 | 0 |  | 8.23 |
| *Tadorna_ferruginea* | 10.9 | 8.5 | 28.5 | A | 66 | 885.22 | 10 | 10 | 0 | 80 | 0 | 100 | 0 | 0 | 133 | 7.63 |
| *Tadorna_tadorna* | 10.9 | 8.9 | 29.5 | A | 62.5 | 1418.68 | 20 | 70 | 0 | 10 | 0 | 90 | 10 | 0 | 121.5 | 7.50 |
| *Tringa_erythropus* | 5.6 | 4 |  | A | 30.5 | 77.5 | 20 | 80 | 0 | 0 | 0 | 80 | 20 | 0 | 64 | 6.87 |
| *Tringa_glareola* | 5.2 | 4 | 22.5 | A | 21 | 158 | 0 | 100 | 0 | 0 | 0 | 50 | 50 | 0 | 55.5 | 7.36 |
| *Tringa_nebularia* | 6.3 | 4 | 24 | A | 32.5 | 71.4 | 20 | 80 | 0 | 0 | 0 | 60 | 40 | 0 | 69 | 7.27 |
| *Tringa_ochropus* | 5.6 | 4 | 21.5 | A | 22.5 | 48.4 | 20 | 70 | 0 | 10 | 0 | 50 | 40 | 0 | 59 | 7.39 |
| *Tringa_stagnatilis* | 5.6 | 4 |  | A | 24 | 129 | 20 | 60 | 0 | 20 | 0 | 60 | 40 | 0 | 57 | 7.16 |
| *Tringa_totanus* | 6.2 | 4 | 25.5 | A | 28 | 62.1 | 20 | 80 | 0 | 0 | 0 | 80 | 20 | 0 | 62.5 | 7.61 |
| *Vanellus_cinereus* | 8.9 | 4 | 28.5 | A | 35.5 | 165 | 0 | 100 | 0 | 0 | 0 | 0 | 100 | 0 | 75.5 | 6.50 |
| *Vanellus_vanellus* | 9 | 4 | 25.5 | A | 29.5 | 198 | 0 | 100 | 0 | 0 | 0 | 0 | 100 | 0 | 84.5 | 7.50 |
| *Xenus_cinereus* | 6.4 | 4 | 23.5 | A | 23.5 | 123 | 0 | 60 | 0 | 40 | 0 | 50 | 50 | 0 | 58 | 7.12 |

**Figure S1** Hypothesized causal structure of the relationships among lake productivity, waterbird functional diversity (FD), geographic variables and local environmental factors, showing the latent variables and indicator variables used in the structural equation models. Direct effects are shown in black arrows and indirect effects are shown in red arrows.


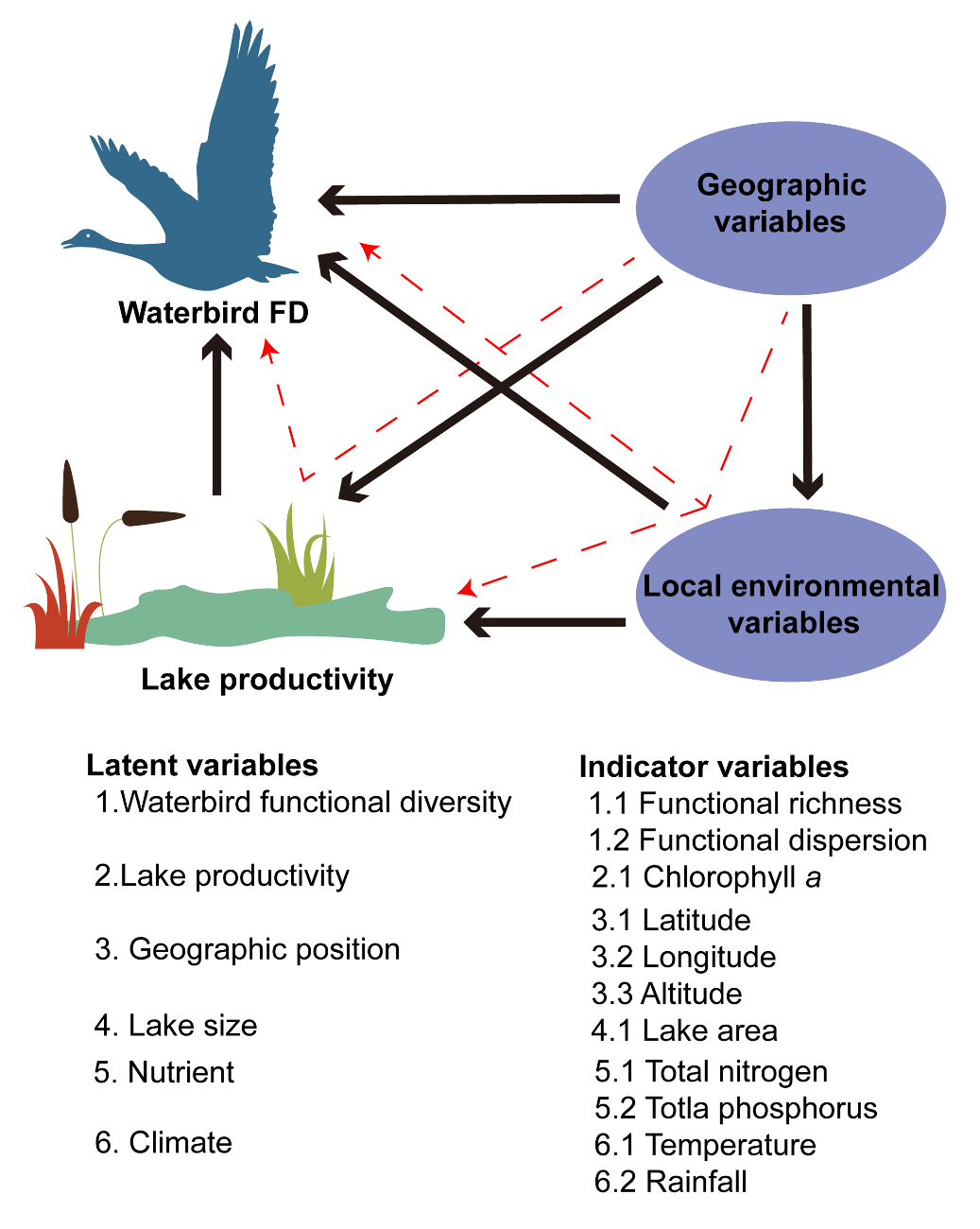


**Figure S2** Map of the CMW (the community-level weighted means of trait values) for the percentage of foraging on ground in the 35 surveyed lakes. Background is the 3D representation of the surface (hillshade).


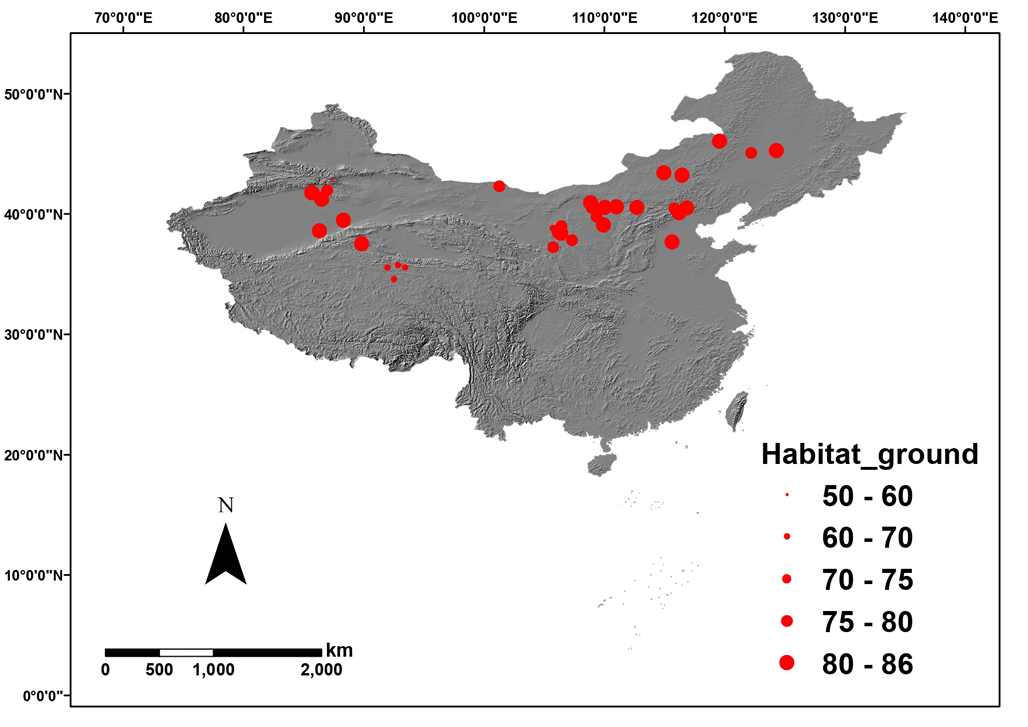


**Figure S3** Map of the CMW (the community-level weighted means of trait values) of percent use of plant materials. Background is the 3D representation of the surface (hillshade).


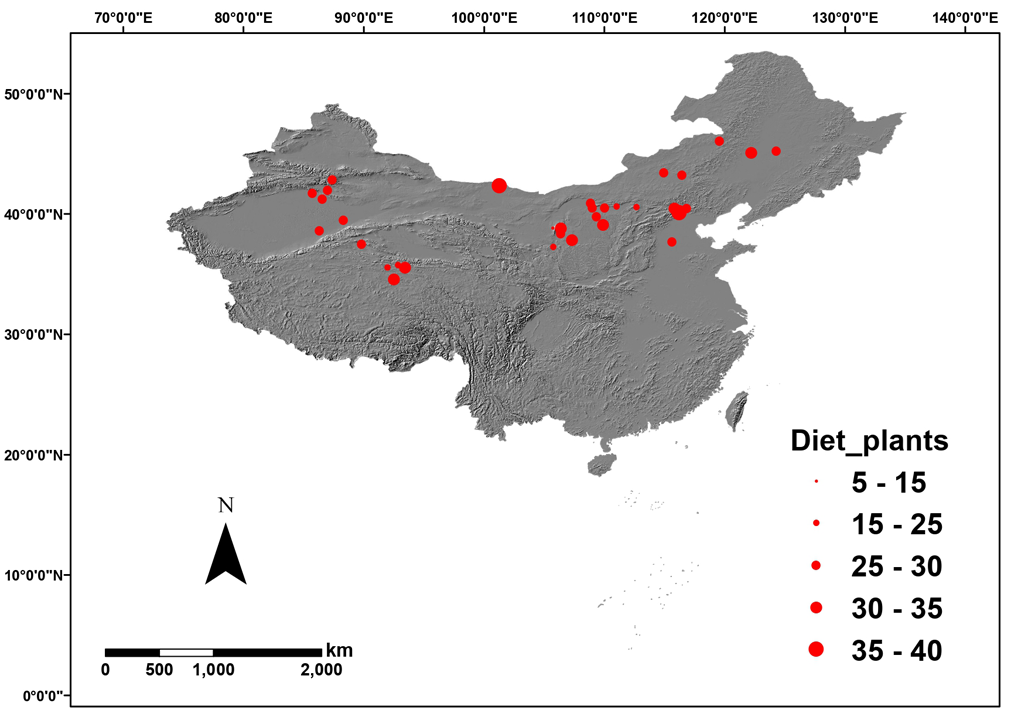

Supplement: Supplementary file 1 — Appendix S1 [file ECE3-10-11237-s001.docx]
